# Exploring the central region of amylin and its analogs aggregation: the influence of metal ions and residue substitutions

**DOI:** 10.3389/fchem.2024.1419019

**Published:** 2024-07-08

**Authors:** Mawadda Alghrably, Giulia Bennici, Gabriela Szczupaj, Noura Alasmael, Somayah Qutub, Batoul Maatouk, Kousik Chandra, Michal Nowakowski, Abdul-Hamid Emwas, Mariusz Jaremko

**Affiliations:** ^1^ Division of Biological and Environmental Sciences and Engineering (BESE), King Abdullah University of Science and Technology (KAUST), Thuwal, Saudi Arabia; ^2^ Faculty of Chemistry, Biological and Chemical Research Centre, University of Warsaw, Warszawa, Poland; ^3^ King Abdullah University of Science and Technology (KAUST), Thuwal, Saudi Arabia; ^4^ Smart Hybrid Materials Laboratory (SHMs), Chemistry Program, Physical Science and Engineering Division, King Abdullah University of Science and Technology (KAUST), Thuwal, Saudi Arabia; ^5^ Core Lab of NMR, King Abdullah University of Science and Technology (KAUST), Thuwal, Saudi Arabia

**Keywords:** human islet amyloid polypeptide (hIAPP), amylin analogues, central region, aggregation, fibril formation, metal ions, copper, zinc

## Abstract

Human amylin (hIAPP) is found in the form of amyloid deposits within the pancreatic cells of nearly all patients diagnosed with type 2 diabetes mellitus (T2DM). However, rat amylin (rIAPP) and pramlintide - hIAPP analogs - are both non-toxic and non-amyloidogenic. Their primary sequences exhibit only slight variations in a few amino acid residues, primarily concentrated in the central region, spanning residues 20 to 29. This inspired us to study this fragment and investigate the impact on the aggregation properties of substituting residues within the central region of amylin and its analogs. Six fragments derived from amylin have undergone comprehensive testing against various metal ions by implementing a range of analytical techniques, including Nuclear Magnetic Resonance (NMR) spectroscopy, Thioflavin T (ThT) assays, Atomic Force Microscopy (AFM), and cytotoxicity assays. These methodologies serve to provide a thorough understanding of how the substitutions and interactions with metal ions impact the aggregation behavior of amylin and its analogs.

## 1 Introduction

Islet Amyloid Polypeptide (IAPP) is a 37-amino acid residue peptide stored as a prohormone within secretory granules inside the β-cells of the pancreas before it is processed to a mature hormone and secreted extracellularly (Brender et al., 2011). Human IAPP (hIAPP) is part of the endocrine pancreatic system that contributes to glycemic control. The peptide is co-stored and co-secreted with insulin in an approximately 100:1 ratio of insulin:amylin from the pancreatic β-cells into the blood circulation (Rorbach-Dolata and Piwowar, 2019). It is released following food intake to regulate blood glucose levels by slowing gastric emptying and promoting the feeling of fullness after the meal, thereby preventing post-prandial spikes in blood glucose levels (Alghrably et al., 2019). This peptide (hIAPP) is aggressively amyloidogenic *and has a* high tendency to form isolate amylin deposits, which are observed particularly in the islets of Langerhans of patients with T2DM, and it has been implicated in the disruption of the cellular membrane of β-cells (Hartman et al., 2009; Wineman-Fisher and Miller, 2017). Various biochemical investigations have demonstrated that the aggregation tendencies of hIAPP can be attributed to its amino acid sequence, particularly the region spanning ^22^NFGAILS^28^, which significantly influences the process of amyloid formation (Emwas et al., 2021; Rhoades et al., 2000). The primary sequence of amylin has been found to be strongly conserved in all mammals studied so far (Westermark et al., 1990). While the IAPP of humans, non-human primates, including cats and the degu, are also known to develop islet amyloid (Westermark et al., 2011; Chiu et al., 2013), it has been discovered that rat amylin (rIAPP) does not. rIAPP amino acid sequence differs from hIAPP in six residues, having three prolines in position 25, 28, and 29, F23L and I26V substitutions. As stated earlier, hIAPP plays an important role in glycemic regulation. However, it cannot be used as a drug for diabetic patients due to the fact that it has a high tendency to misfold and form cytotoxic fibrils (Westermark et al., 2011), a process that itself is strongly associated with β-cell degeneration in T2DM (Phillips and Horowitz, 2006; Hartman et al., 2009; Cao et al., 2013). This limitation led to the development of amylin synthetic analogue, pramlintide, to overcome the physicochemical properties of aggregation and toxicity (Magrì et al., 2020). The amino acid sequence of pramlintide is characterized by the substitution of Ala25, Ser28, and Ser29 in the hIAPP sequence with prolines (Pittner et al., 1994). Similar to rIAPP, these substitutions eliminate the ability of the compound to form amyloidogenic fibrils, making pramlintide a highly suitable treatment for diabetes (Roth et al., 2008). Pramlintide is commonly used in the United States in insulin-resistant patients (Ryan et al., 2005) and has also recently been shown to have antimicrobial properties (Wang et al., 2012).

It is important to highlight that experiments conducted on hIAPP have established that the residues within this central region are responsible for conferring amyloidogenic properties to the peptide, thus making it capable of independently forming fibrils (Chiu et al., 2013; Frigori, 2017; Bhowmick et al., 2022). With this perspective in mind, our work focuses on investigating the impact of substituting residues within the central region of amylin and its analogs on their aggregation properties (Buchanan et al., 2013). Fragments derived from human amylin, rat amylin, and pramlintide, along with three other custom-designed fragments originating from each of these mentioned peptides (Table 1), have undergone comprehensive testing against various metal ions. Metal ions are recognized for enhancing the formation of amyloids and introducing novel electrochemical and spectroscopic characteristics to amyloid materials (Ward et al., 2008a; Brender et al., 2010; Rivillas-Acevedo et al., 2015) Furthermore, the interaction of metals with amyloids markedly impacts their structure and morphology (Salamekh et al., 2011; Rowińska-Zyrek, 2016a; Atrián-Blasco et al., 2018; Abdelrahman et al., 2020). To dissect their influence on these amylin fragments, we have employed a combination of analytical techniques, including Nuclear Magnetic Resonance (NMR) spectroscopy, Thioflavin T (ThT) assays, Atomic Force Microscopy (AFM), and cytotoxicity assays. These methodologies serve to provide a thorough understanding of how these substitutions and interactions with metal ions impact the aggregation behavior of amylin and its analogs (Poulson et al., 2019; Abdelrahman et al., 2023; Roy et al., 2023). Our findings with ThT assays revealed enhanced aggregation of hIAPP-F, influenced by the addition of Zn(II). While Cu(II) significantly decreased the aggregation of the hIAPP-F. On the other hand, Pramlintide-M and Pramlintide-M:Zn(II) showed a slight increase in fluorescence intensity. AFM analysis confirmed fibril formation for hIAPP-F and Pramlintide-M. Specifically, the hIAPP-F sample displayed elongated, twisted fibrils consistent with matured amyloid structures and a similar fibril morphology is also evident in the hIAPP-F:Zn sample. While Pramlintide-M samples revealed helical fibrils and spherical aggregates with smoother surfaces compared to hIAPP. The fibril of Pramlintide-F morphology is appearing as a helical structure. NMR spectroscopy corroborated accelerated aggregation in the presence of Zn(II) for Pramlintide-M peptide. Cytotoxicity assays on HeLa cells indicated reduced viability for hIAPP-F and Pramlintide-M, mitigated by peptide-Cu(II) complexes but unexpectedly increased by peptide-Zn(II) complexes. These findings shed light on the amyloidogenic nature of amylin peptides and provide insights into amyloid formation mechanisms.

**TABLE 1:** Summary of full-length, fragmented, and mutated variants of the different amylin peptide analogues, including primary sequence, Molecular Weight (MW), and Isoelectric Point (IP).

Peptide	Primary sequence	MW(g/mol)	IP
hIAPP (Full-length)	KCNTATCATQRLANFLVHSSNNFGAILSSTNVGSNTY-NH_2_	3903.28	8.90
rIAPP (Full-length)	KCNTATCATQRLANFLVRSSNNLGPVLPPTNVGSNTY-NH_2_	3920.6	9.50
Pramlintide (Full-length)	KCNTATCATQRLANFLVHSSNNFGPILPPTNVGSNTY-NH_2_	3951.4	8.90
hIAPP-F	SNNFGAILSS	1,009	5.24
rIAPP-F	SNNLGPVLPP	1,007.15	5.24
Pramlintide-F	SNNFGPILPP	1,055.2	5.24
hIAPP-M	SNNFGAILPP	1,029.16	5.24
rIAPP-M	SNNLGPILPP	1,021.18	5.24
Pramlintide-M	SNNFGPILSS	1,035.12	5.24

## 2 Materials and methods

### 2.1 Peptides

Table 1 lists the abbreviations, sequences, and molecular weights for the studied peptides used in this chapter. These peptides were purchased from KareBayBiochem, GeneScript (certified purity of 99.30% and >95%) used without further purification.

Cu(NO_3_)_2_ and ZnCl_2_ were purchased from Fisher Scientific.

### 2.2 Thioflavin-T (ThT) assay

Preparation of IAPP fragments solutions and thioflavin-T aggregation assays were performed as described earlier (Alghrably et al., 2020). In brief, IAPP fragments were dissolved in 50 mM phosphate buffer to concentrations of 1 mM. Stock solutions were diluted to the desired concentrations, and samples were used fresh each time. It is important to mention that due to the observation of turbidity after dissolving hIAPP-F and Pramlintide-M, both peptides were sonicated for 25 min. All of the aggregation reactions were conducted at 25°C with 40 μM IAPP fragments/mutants (final concentration) in 50 mM phosphate buffer (pH 7.4), and 20 μM thioflavin T in a final volume of 200 μL in each of the 96-well flat-bottom black plate (Corning 3915). We tested the aggregation of each IAPP fragment/mutants alone and with the addition of two metal ions Cu(II) and Zn(II). The metal ions were added to the IAPP fragments in 1:1 ratio. The plate was sealed with transparent film (Duck Brand Crystal Clear Tape, Avon, OH). Thioflavin T fluorescence emission at 528/20 nm (excitation at 485/20) was monitored on a Cytation 5 Multi-Mode Microplate. The measurements were collected by top reading each 5 min after a 3 s shake with an extended gain.

### 2.3 Atomic force microscopy (AFM)

AFM imaging was conducted in a 256-scanning line at a 1 Hz scanning rate by using Bruker’s Nano Dimension Icon (Bruker Nano Inc., Germany). In this procedure, 200–300 μM hIAPP-F and Pramlintide-M were incubated with and without selected metal ions at 25°C for 24 h. All the samples were dissolved in 50 mM phosphate buffer (pH 7.4). The height of peptide aggregates was obtained from its section tool through Nano Scope Analysis 1.5, and the line position with average effects in the images was selected, except peptides alone in fibril form.

### 2.4 Nuclear magnetic resonance (NMR)

All peptides (except hIAPP-F and Pramlintide-M) were dissolved in 99.8% deuterium oxide containing 20 mM of phosphate buffer (pH 7.4). All samples contained 1.5 mM peptide concentration in 600 μL of sample volume except for hIAPP-F and Pramlintide-M, for which the final concentration was 100 μM (both peptides have a high tendency to aggregate at room temperature). The samples’ pH was manually adjusted to 7.4 with 0.1 M NaOD stock solution in 100% D2O. These samples were used for the ^1^H and ^13^C resonances assignment based on 2D spectra: ^1^H-^13^C HSQC (Bodenhausen and Ruben, 1980) tuned for the aliphatic region, HSQC-TOCSY (Koz´min´ski and Koz´min´ski, 1999), ^1^H-^1^H TOCSY (Braunschweiler and Ernst, 1983) mixing time 20 ms and 80 ms, ^1^H-^1^H ROESY (Davis, 1985) mixing time 300 ms and 500 ms. During the hIAPP-F and Pramlintide-M sample preparation, a temperature of 90°C was used to dissolve the peptides. After that, both samples were sonicated during the cooling down process to obtain a homogeneous solution. Spectra were measured immediately after sample preparation. The samples with the addition of metal ions were prepared with the same method as the samples of high tendency aggregation peptides hIAPP-F and Pramlintide-M.

All experiments were performed at a temperature of 310 K. The NMR spectra were acquired on spectrometers of 950, 800, and 700 MHz Bruker Avance NEO equipped with cryogenic TCI probes:

• rIAPP-F, hIAPP-F, Pramlintide-M: 700 MHz, ^1^H frequency = 700.21 MHz, ^13^C frequency = 176.07 MHz
• rIAPP-M: 800 MHz, ^1^H frequency = 800.18 MHz, ^13^C frequency = 201.21 MHz
• Pramlintide-F, hIAPP-M: 950 MHz, ^1^H frequency = 950.30 MHz, ^13^C frequency = 238.96 MHz

Data was processed in TopSpin Bruker software (Bruker, 2024) and analyzed with NMRFAM Sparky software (Lee et al., 2015).

Analysis of ^1^H and ^13^C resonances was performed applying a standard procedure (Wüthrich, 1986) by the inspection of 2-dimensional spectra measured for peptides rIAPP-F, Pramlintide-F, hIAPP-M, and rIAPP-M.

### 2.5 Cytotoxicity assay

The cytotoxicity of the different peptides (rIAPP-F, hIAPP-F, Pramlintide-F, hIAPP-M, rIAPP-M, Pramlintide-M, and hIAPP) was tested using CCK-8 assay according to the manufacturer’s protocol. Briefly, cervical carcinoma cells (HeLa) were seeded into 96 well plates 8 × 10^3^ cells/well in 100 µL DMEM culture medium with 10% FBS and 1% Penicillin-Streptomycin and incubated in a 5% CO2 incubator at 37°C overnight. After that, the cells were incubated with the different peptides prepared in deionized water at concentrations (200, 400, 600, and 800 μg/mL) for 24 h. In addition, the cytotoxicity of hIAPP-F and Pramlintide-M prepared in 0.5 mg/mL Cu(NO_3_)_2_ and ZnCl_2_ solutions at the same concentrations (200, 400, 600, and 800 μg/mL) was also tested. Cells without any treatment were used as a control, and all the conditions were done in triplicates. After 24 h, the media was discarded from the plate, and cells were washed with PBS. Then, 10 µL of CCK-8 and 90 µL of fresh DMEM media were added to each well and incubated for 4 h. The absorbance was measured at OD = 570 nm and 605 nm using a microplate spectrophotometer (BioRad-xMark Microplate Absorbance Spectrophotometer). The ratio of the absorbance at 570/605 was calculated, and the average absorbance value of wells without cells was considered as blank and subtracted from all wells to cancel the background noise. The cells without any treatment were used as a control, and their mean value was considered 100% viable. The other treated cells’ viability was calculated using the formula: Cells viability % = (Corrected Mean Absorbance of treated cells)/(Corrected Mean Absorbance of control) ×100.

## 3 Results and discussion

### 3.1 The effect of metal ions on the aggregation kinetics of IAPP fragments

The Thioflavin T (ThT) assay is a widely employed method for assessing the dynamics of protein assembly in a controlled laboratory setting. ThT, a fluorescent dye, quickly binds to the β-sheet structures found within aggregated fibrils, leading to a noticeable increase in fluorescence emission. This alteration in fluorescence intensity isn't solely influenced by the concentration of fibrils but also by the shift in the equilibrium between two ThT binding sites on a given fibril surface (Gade Malmos et al., 2017).

**FIGURE 1:**
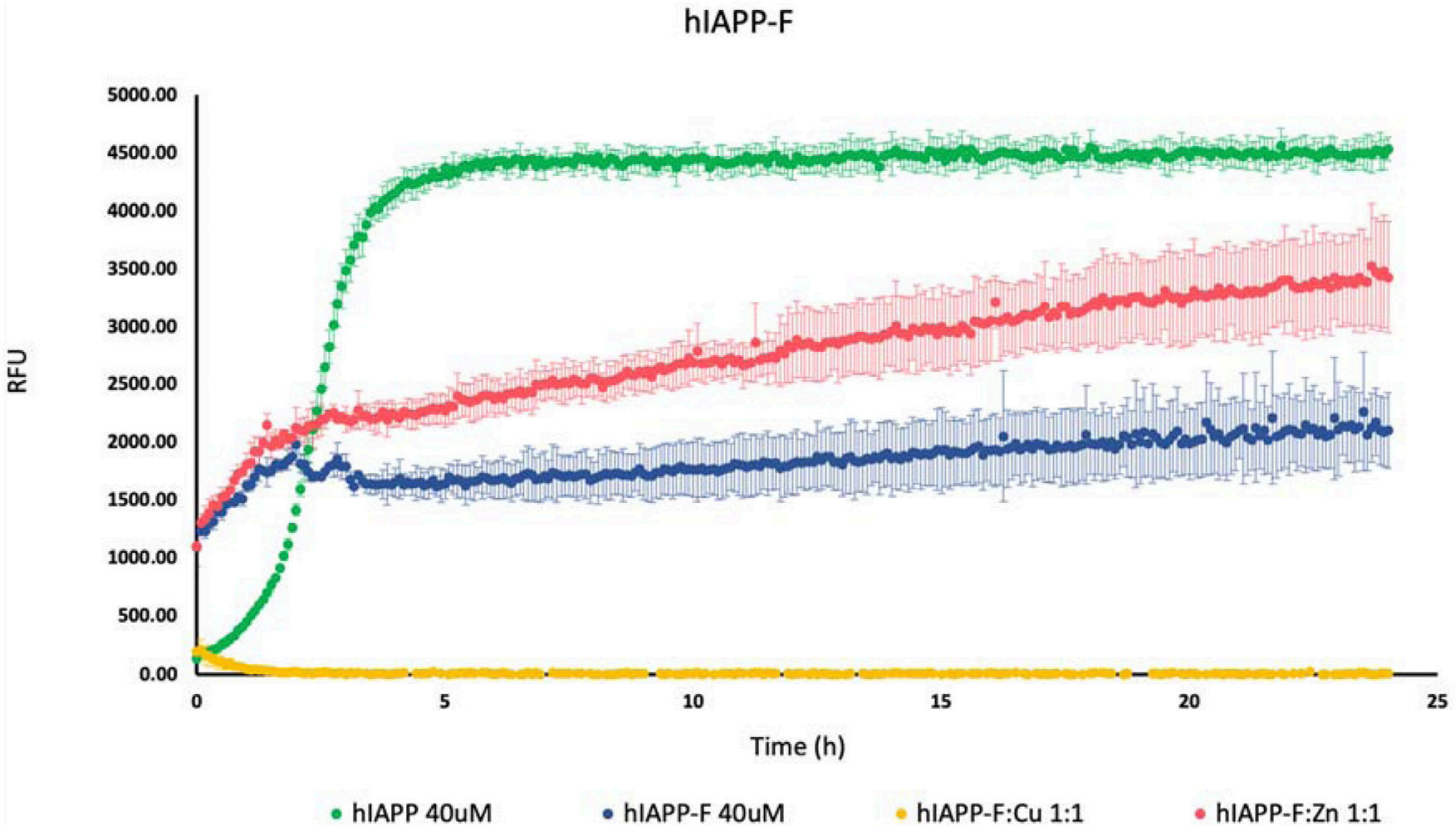
Effects of Cu(II) and Zn(II) on hIAPP-F aggregation in a phosphate-buffered solution. Amylin peptides (40 μM) were subjected to aggregation at 25°C in the presence of 20 μM ThT and 50 mM phosphate buffer pH 7.4.

To investigate how Cu(II) and Zn(II) influence the aggregation of IAPP peptide fragments and mutants, we assessed their aggregation behavior both with and without the presence of these specific metal ions. These experiments were conducted in a phosphate buffer with a pH of 7.4, and we monitored the aggregation process by measuring ThT fluorescence. Controls of Cu(NO_3_)_2_ and ZnCl_2_ were used (Supplementary Figure S1). As a positive control, we used hIAPP (full length), which is known for its propensity to rapidly aggregate (da Silva et al., 2016; Reddy et al., 2010). As expected, we observed an increase in the fluorescence intensity of human amylin fragment (hIAPP-F) (Figure 1). This is attributed to the fact that residues 20–29 are crucial for the amyloid formation of hIAPP, and this fragment is recognized as the amyloidogenic region (Cao et al., 2020; Wang et al., 2022; Lin et al., 2023). It is clear from the ThT graph that hIAPP-F and hIAPP-F:Zn(II) solutions had aggregated in the same pattern and that the aggregation trends of both solutions were lower than the full-length-hIAPP solution (Figure 1). Moreover, the elongation phase of hIAPP-F and hIAPP-F:Zn(II) solutions started rapidly, which indicates the fast addition of monomers to growing fibrils. Nevertheless, it was shorter in time compared to the same phase of the hIAPP (full-length) solution. Both curves of hIAPP-F and hIAPP-F:Zn(II) reached the saturation phase after ∼2.5 h. Comparing the fluorescence intensity of hIAPP-F with hIAPP-F:Zn(II), we observed that the presence of Zn(II) in the solution of hIAPP-F influenced the aggregation and increased the fluorescence intensity. In contrast, the addition of Cu(II) to the hIAPP-F solution significantly decreased the fluorescence intensity and completely inhibited the aggregation of hIAPP-F. In line with previous research, our study demonstrated that Zn(II) ions indeed influence the aggregation behavior of hIAPP-F, as evidenced by the increase in fluorescence intensity observed in our ThT assay. This finding is consistent with the work of D. Łoboda and M. Rowińska-Żyrek (Rowińska-Zyrek, 2016b; Łoboda and Rowińska-Żyrek, 2017) who highlighted the role of Zn(II) in promoting the formation of large Zn(II)-amylin aggregates. Our results further support these observations, as the presence of Zn(II) in the hIAPP-F solution accelerated the aggregation process, leading to a faster onset of the elongation phase and ultimately reaching saturation earlier compared to the control solution without Zn(II). This suggests a direct interaction between Zn(II) ions and amylin, influencing the kinetics of fibril formation. Additionally, the contrasting effect of Cu(II) observed in our study (ThT results), where it inhibited aggregation and decreased fluorescence intensity, are consistent with the observations made by Benjamin Ward et al., (Ward et al., 2008b), who demonstrated that Cu(II) effectively inhibits amylin aggregation and prevents the formation of β-sheet conformers.

**FIGURE 2:**
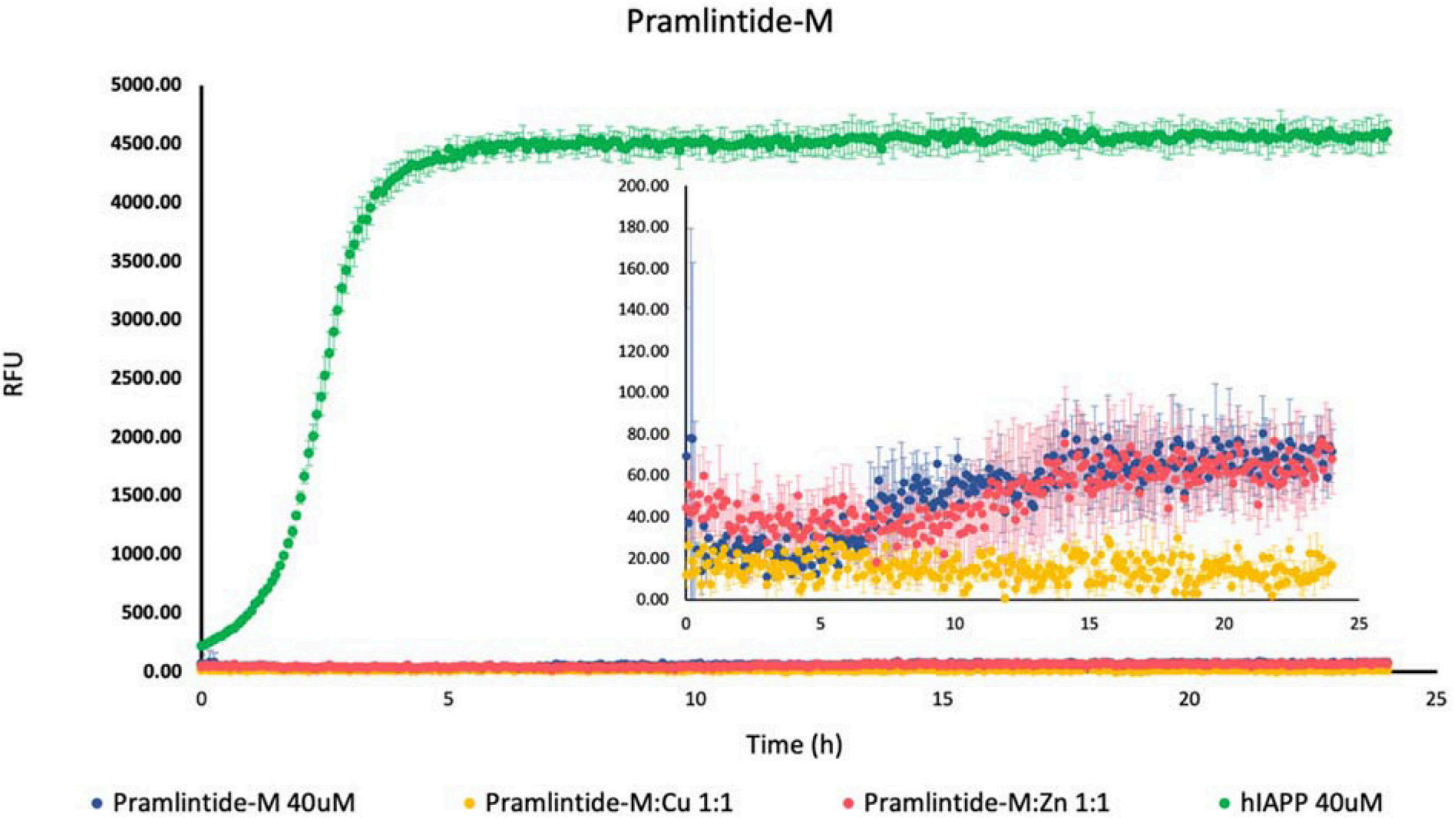
Effects of Cu(II) and Zn(II) on Pramlintide-M aggregation in a phosphate-buffered solution. Amylin peptides (40 μM) were subjected to aggregation at 25°C in the presence of 20 μM ThT and 50 mM phosphate buffer pH 7.4.

On the other hand, there was a slight increase in the fluorescence intensity with Pramlintide-M and Pramlintide-M:Zn(II) (Figure 2). The peptide alone started to aggregate after 7 h, while the aggregation of the solution containing Pramlintide-M:Zn(II) started after 12 h. Both solutions reached the saturation state after 14 h. The increase in the fluorescence intensity can be due to the mutation of the last two prolines at positions 9 and 10 of the sequences with serins within the primary sequence of the short peptide, while the first proline at position six remained intact. This confirms that the presence of proline residues within the sequence of the peptide inhibits the β-sheet formation, thus decreasing aggregation. Nevertheless, the substitution of the last two prolines with serins did not completely inhibit the aggregation. However, the aggregation rate was very low compared to hIAPP-F, which doesn’t contain proline residues within its sequence. Thus, proline 6 (corresponding to proline 25 within amylin full length and its studied analogs) plays an important role in the inhibition of the aggregation. Last but not least, the addition of Cu(II) to the Pramlintide-M solution inhibited the aggregation.

rIAPP-F, pramlintide-F, hIAPP-M, and rIAPP-M did not show any increase in the fluorescence intensity (Supplementary Figure S2). Moreover, neither metal ions Cu(II) nor Zn(II) have any influence on the aggregation rate of these four peptides (Supplementary Figure S3). This can be explained by the presence of proline in each of their primary sequence. However, it is noteworthy that hIAPP-M has only the last two serins mutated with prolines, and there was no aggregation activity with this peptide. This could indicate that the presence of the serins is a crucial factor in the β-sheet formation because of the presence of the -OH group that can easily be involved in the hydrogen bond network formation. Moreover, it is well known that the presence of the prolines within the sequence does not favor the aggregation. It seems the proline residues can expose their full potential for being a beta-sheet breaker when placed within an amino acid sequence as well as at their end.

### 3.2 Probing protein structures and interactions: NMR insights into aggregating peptides and metal ion complexes

Nuclear Magnetic Resonance (NMR) has been employed in the examination of diverse functional compounds, including natural products, and has evolved into a pivotal technology acknowledged as the “gold standard” in medical and pharmacological investigations (Emwas et al., 2020).

It is the preferred technique for examining both the structures and dynamics of proteins and for exploring interactions between proteins and metal ions (Alsiary et al., 2020a; Alsiary et al., 2020b).

**FIGURE 3:**
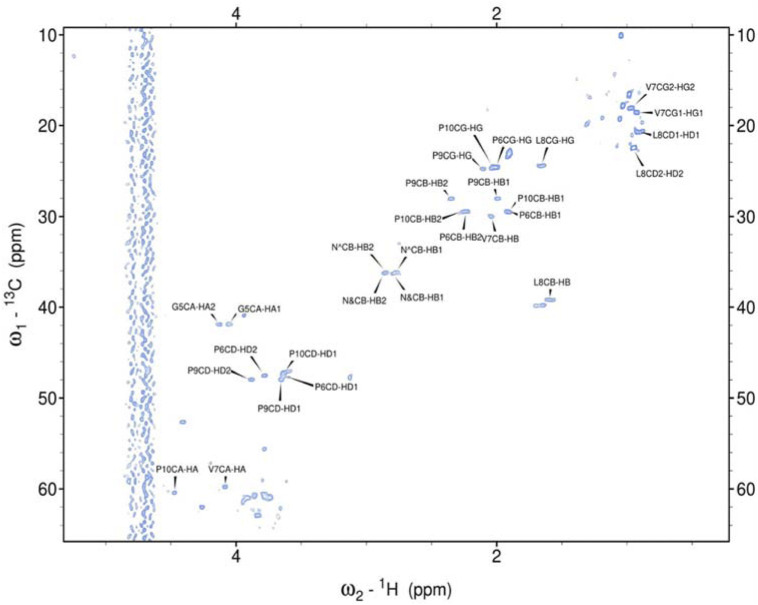
Assigned spectrum ^1^H-^13^C HSQC of rIAPP-F.

**FIGURE 4:**
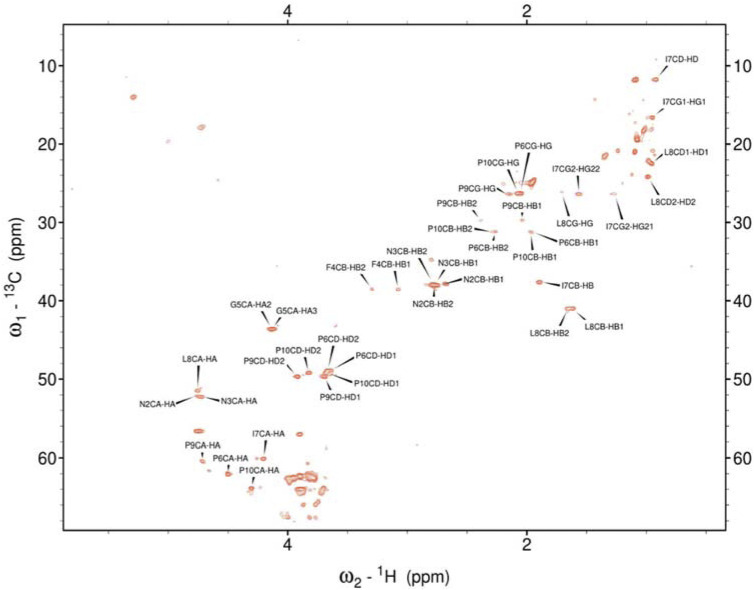
Assigned spectrum ^1^H-^13^C HSQC of Pramlintide-F.

**FIGURE 5:**
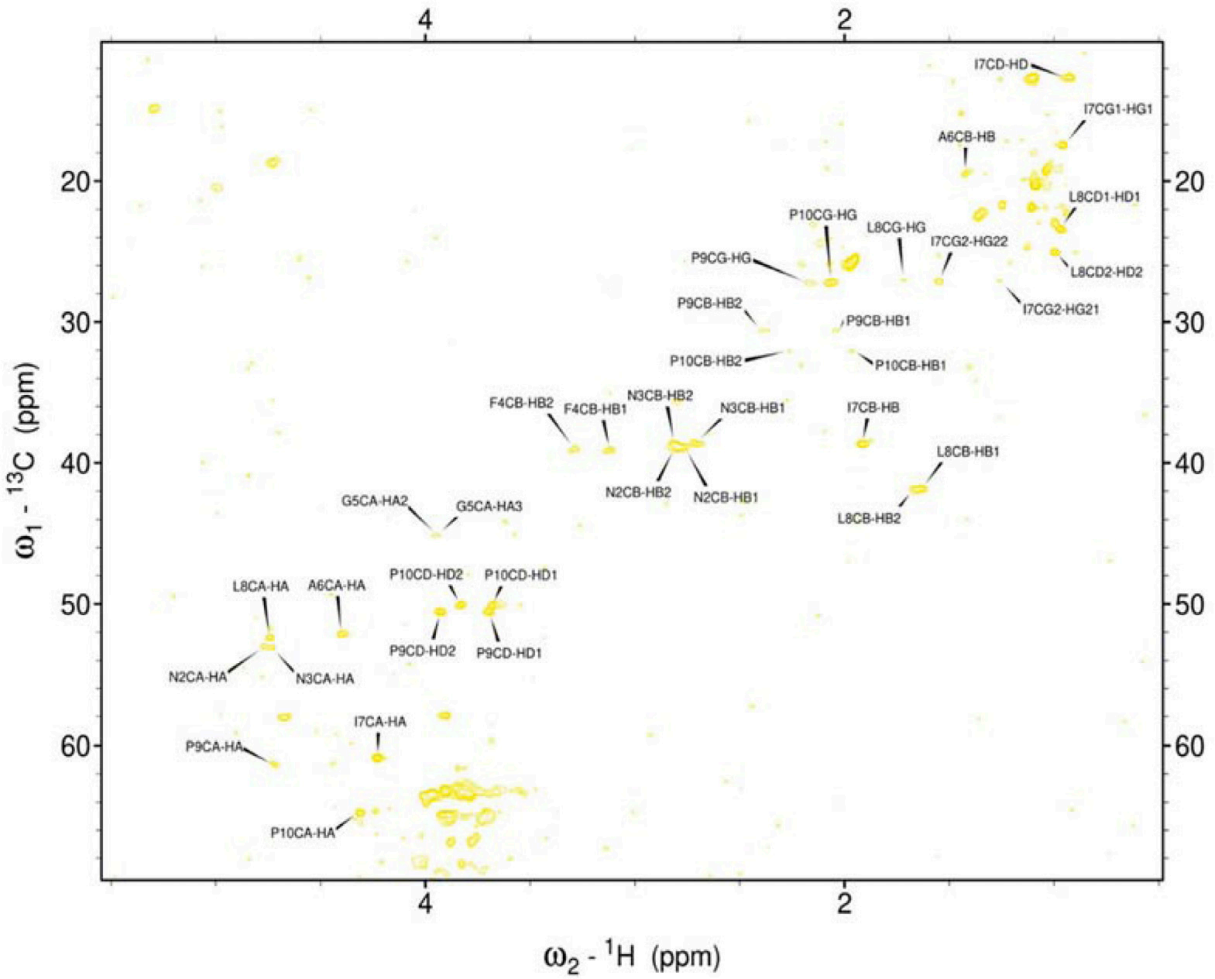
Assigned spectrum ^1^H-^13^C HSQC of hIAPP-M.

**FIGURE 6:**
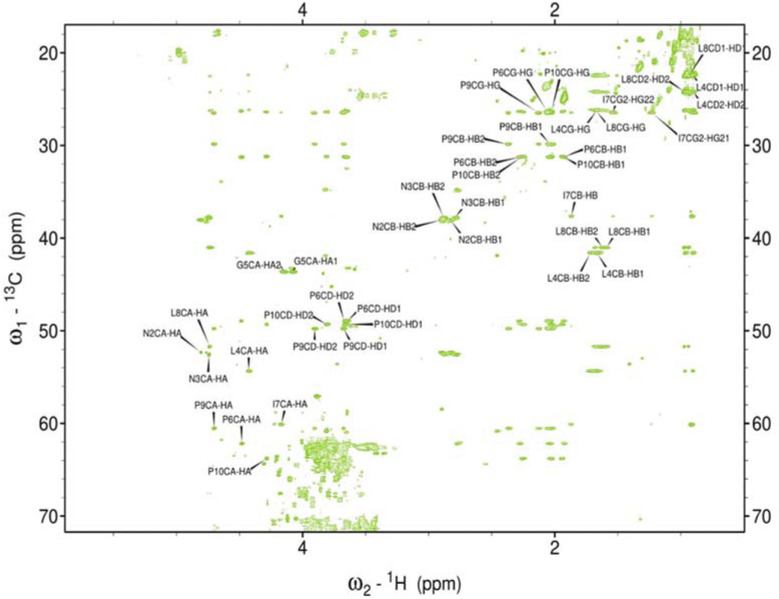
Assigned spectrum HSQC-TOCSY of rIAPP-M.

Hence, it was employed to investigate the amino acids involved in the aggregation and the binding mode of the metal ion complexes formed with the studied peptides. Due to rapid changes occurring in the two aggregating peptides at high concentrations, only one-dimensional spectra were recorded. Subsequently, 2D NMR spectra were obtained and assigned for the peptides rIAPP-F, pramlintide-F, hIAPP-M, and rIAPP-M (Supplementary Table S1; Supplementary Table S2; SI) (Figures 3–6). Assignments from the two-dimensional spectra of the four non-aggregating peptides were transferred to their corresponding one-dimensional spectra. Furthermore, by identifying peaks for each residue based on chemical shifts assigned for non-aggregating peptides (Supplementary Figures S6–S9), the one-dimensional spectra of aggregating peptides hIAPP-F and pramlintide-M could be assigned (Supplementary Figures S10, S11). Analysis of the spectra revealed that recognition of the residues involved in aggregation and determination of the binding mode of Zn(II) complexes with hIAPP-F is not possible due to a lack of specific changes in chemical shifts after the addition of metal ions (Supplementary Figure S4).

As the peptides rIAPP-F, pramlintide-F, hIAPP-M, and rIAPP-M have a low tendency to aggregate, no signal loss was observed over time. The situation differs with hIAPP-F and pramlintide-M. The signal intensity of both peptides decreased over time. These changes can be attributed to the aggregation process, which perfectly agrees with the ThT and atomic force microscopy (AFM) results obtained for these molecular systems. As expected for small aggregating peptides, the decreasing intensity of signals over time was equal for each peak, which prevents identifying specific residues involved in the aggregation process. Moreover, the signal intensity deterioration of hIAPP-F occurred faster in the presence of Zn(II), which aligns with the results obtained by other methods (Supplementary Figure S5).

### 3.3 AFM analysis of fibril morphology: investigating aggregating peptides hIAPP-F and Pramlintide-M

**FIGURE 7:**
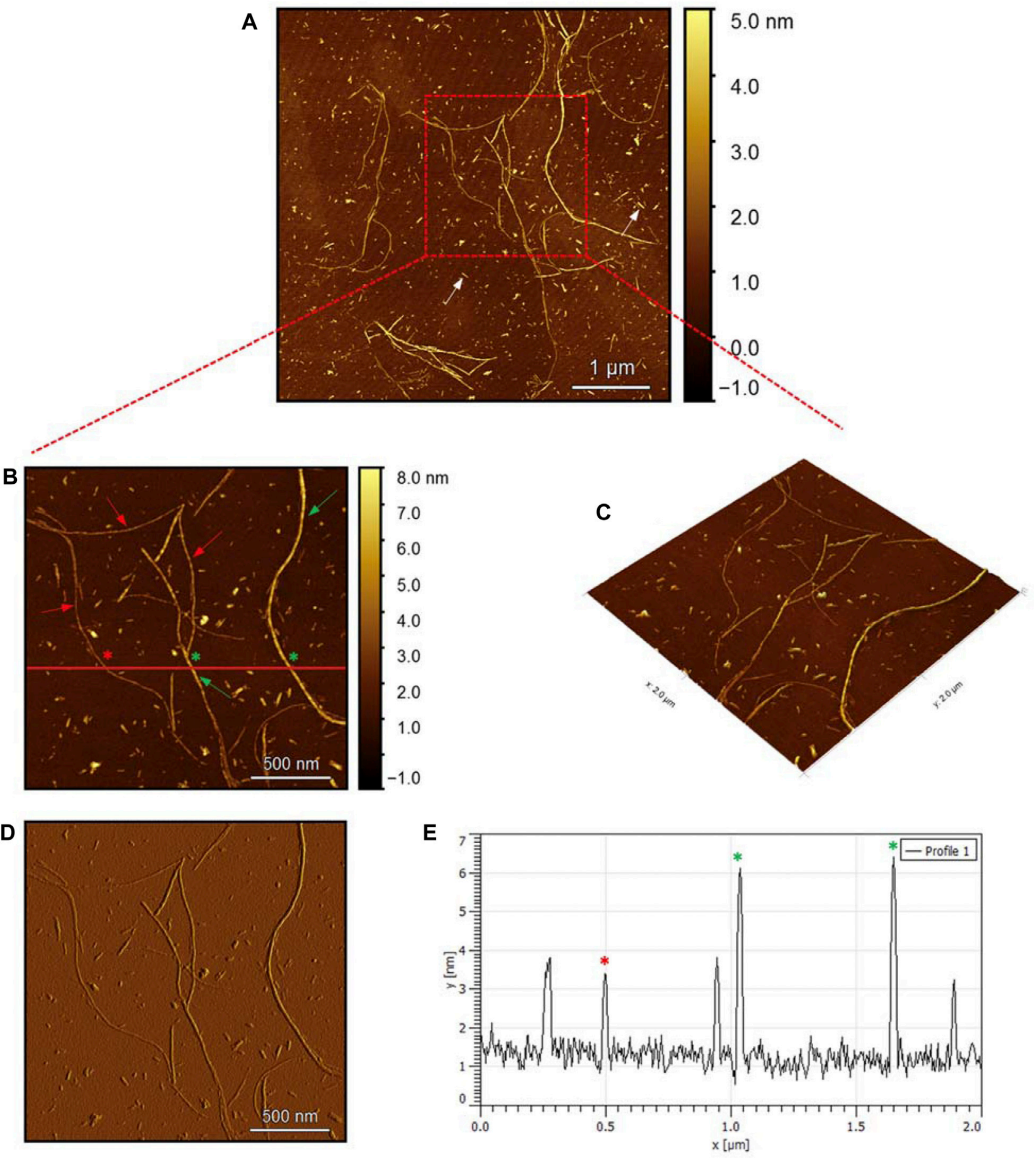
Solid-state AFM images of hIAPP 200 µM sample dissolved in phosphate buffer pH 7.4. Taken after 24 hrs **(A)** Scan area of the formed fibrils. **(B)** 2D and **(C)** 3D views of a magnified ×22 µm area and **(D)** the corresponding stiffness map of B **(E)** the corresponding profile of the diameter section analysis of **(B)**.

**FIGURE 8:**
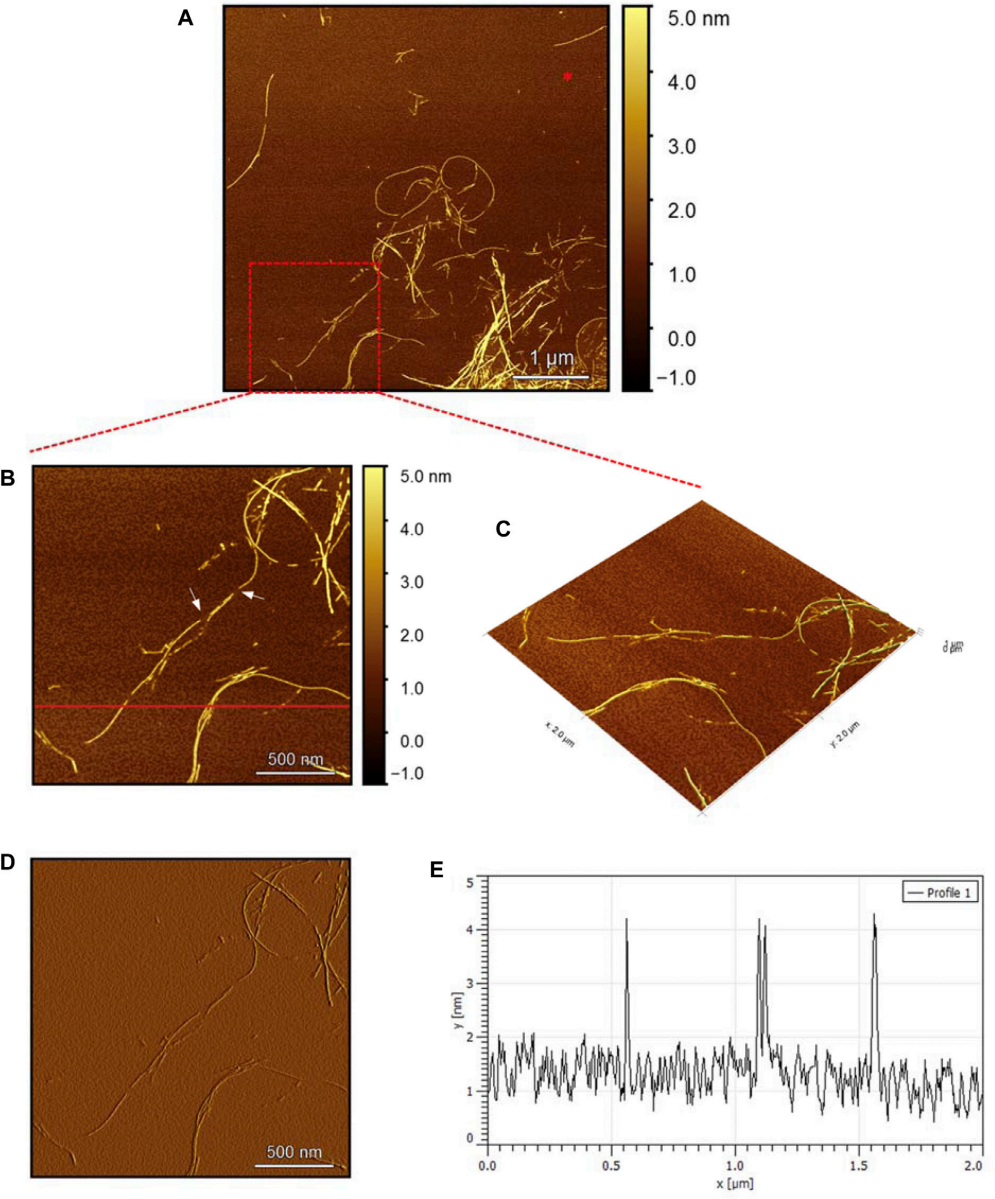
Solid-state AFM images of hIAPP:Cu 200 µM 1:1 ratio sample dissolved in phosphate buffer pH 7.4. Taken after 24 hrs **(A)** Scan area of the formed fibrils. **(B)** 2D and **(C)** 3D views of a magnified ×22 µm area **(D)** the corresponding stiffness map of B **(E)** the corresponding profile of the diameter section analysis of **(B)**.

To evaluate the morphology of the fibril species formed by the two aggregating peptides hIAPP-F and Pramlintide-M, AFM was conducted. The AFM images were taken after 24 h of incubation to allow the fibrils to reach the final stage of the fibrillation process. hIAPP was used as a control sample (Figure 7), to compare the structural morphology of the formed fibrils with the short peptide fragments with a high tendency to aggregate hIAPP-F and Pramlintide-M. In addition to the control, the hIAPP:Cu(II) sample was prepared and examined under the AFM (Figure 8) in order to analyze the effect that Cu(II) introduces in the structural morphology of hIAPP (full length) and compare it with the short peptide with the addition of Cu(II) to the sample. The fibrils that are observed in the control sample of hIAPP vary in length, with some extending beyond 2 µm (Figure 7C). However, they are relatively short worm-like fibrils distributed all over the sample (Figure 7A, represented by the white arrows). These short-length fibrils could be attributed to the formation of more nuclei, resulting in a higher number of fibrils at elevated hIAPP concentrations. The magnified image (Figure 7B) reveals the structural morphology of the formed fibril in more specific detail. There are two distinct types of fibrils (Figure 7B shown with the different colored arrows). The red arrows point to a thinner fibril compared to the other type, denoted by the green arrows. Furthermore, this thinner fibril exhibits a ribbon-like and smoother surface, as evidenced by the contrast variation in the stiffness profile (Figure 7D). The green arrows, on the other hand, indicate a helical fibril formation from the twisting of two-stranded ribbon-like fibrils (Figure 7B). The diameter of the fibrils formed in the control sample can be determined using the cross-section profile chart (Figure 7E), which also shows a variation between the ribbon-like and the helical-structured fibrils (Figures 7B, E represented by the red and green asterisks). The ribbon fibrils of hIAPP, which is indicated in red asterisks, have a diameter of around 2 nm, while the helical fibrils (green asterisks) are approximately 5–6 nm in diameter. On the other hand, in the hIAPP:Cu(II) sample at a 1:1 ratio of 200µM, shorter fibrils are evident (Figure 8C). The average length of these fibrils is less than 2 µm, and they exhibit a unique looping structure (Figure 8A) with intermittent cut sections along the length of the extended fibril (Figure 8B, represented by the white arrows). Moreover, the stiffness profile along the formed fibril, which can be seen in (Figure 8D) shows that the hIAPP:Cu(II) sample exhibits a smoother surface compared to the control sample of hIAPP (Figure 7D). This observation suggests a change in the morphology of fibril formation with the addition of Cu(II). The diameter of the fibrils is reduced compared to the helical fibrils formed in the hIAPP control sample. Nevertheless, it is similar to the ribbon-structured fibrils of hIAPP, measuring approximately 2–3 nm, as determined from the cross-sectional profile chart (Figure 8E). These measurements suggest that these fibrils still did not reach their fully matured form, which is the helical structure. Thus, Cu(II) might slow down or prevent the process of fibril formation.

**FIGURE 9:**
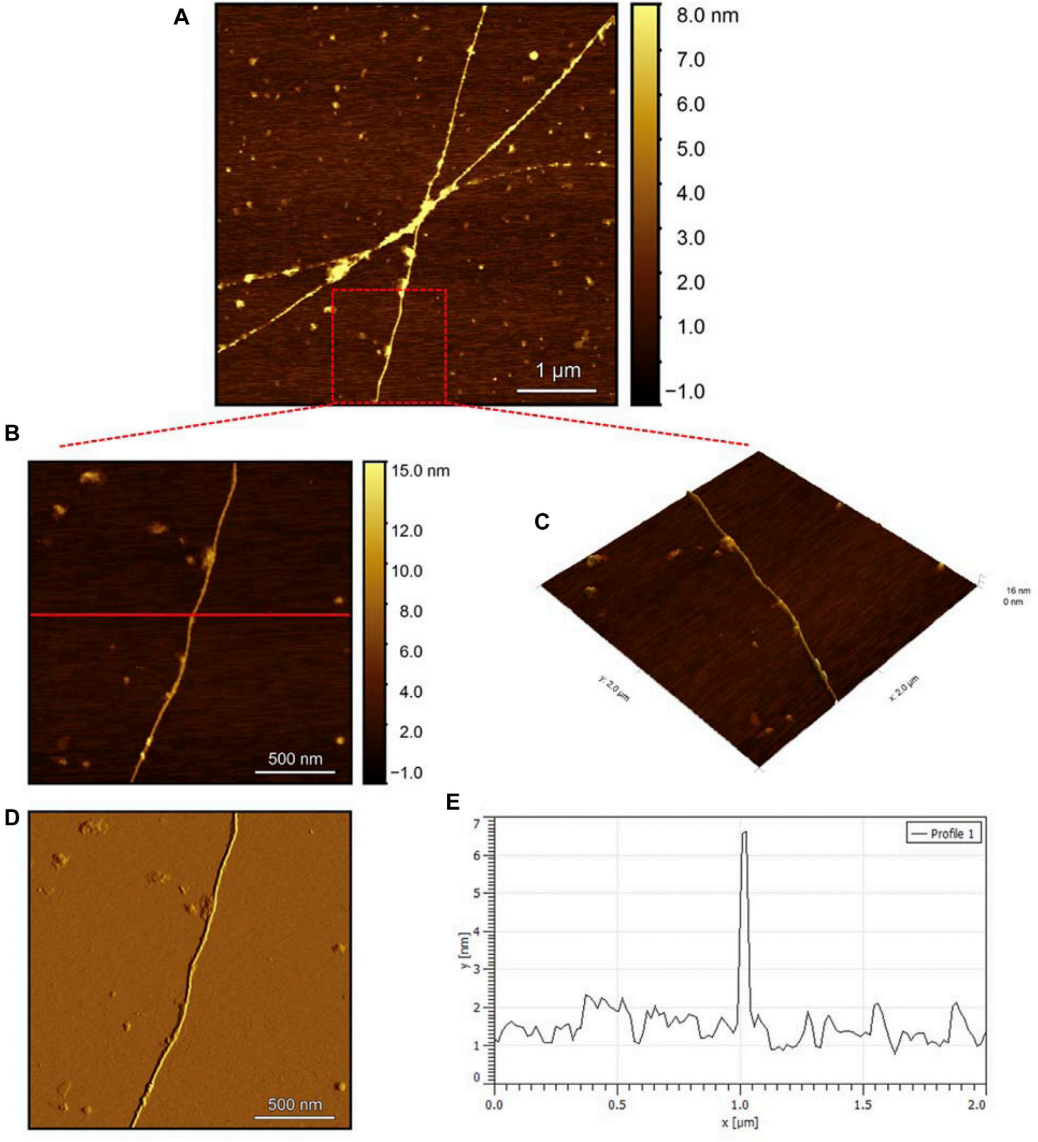
Solid-state AFM images of hIAPP-F 200 µM sample dissolved in phosphate buffer pH 7.4. Taken after 24 hrs **(A)** Scan area of the formed fibrils. **(B)** 2D and **(C)** 3D views of a magnified ×22 µm area **(D)** the corresponding stiffness map of B **(E)** the corresponding profile of the diameter section analysis of **(B)**.

**FIGURE 10:**
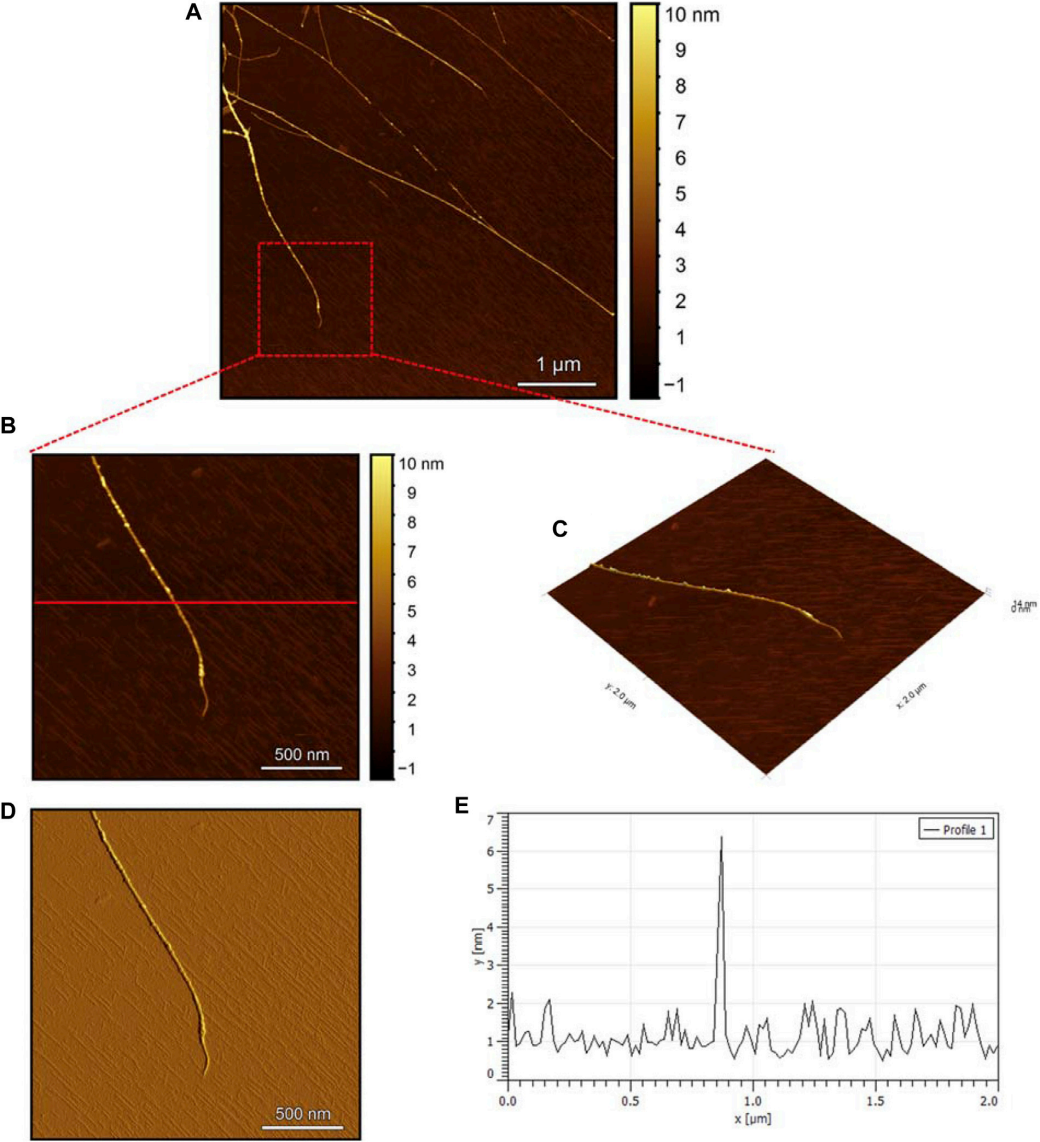
Solid-state AFM images of hIAPP-F:Zn 200uM 1:1 ratio sample dissolved in phosphate buffer pH 7.4. Taken after 24 hrs **(A)** Scan area of the formed fibrils. **(B)** 2D and **(C)** 3D views of a magnified ×22 µm area **(D)** the corresponding stiffness map of B **(E)** the corresponding profile of the diameter section analysis of **(B)**.

The AFM images of the hIAPP-F sample (Figure 9A) reveal the presence of elongated fibrils with a diameter of approximately 6 nm, as indicated by the profile analysis image (Figure 9E). The morphology of these fibrils can be identified by the shape of the species in the magnified images (Figures 9B–D), which is clearly a twisted fibril. The study of Shuai Zhang and others on hIAPP_20–29_ provides detailed insights into the distinction between the immature ribbon-like structure and the final-stage twisted fibrils (Zhang et al., 2013). The type of fibril observed in Figure 9 represents the last stage of the amyloid self-assembly process and is considered to be mature due to its structural complexity (Adamcik et al., 2010; Lara et al., 2011). This twisted fibril morphology aligns with what has previously been observed for hIAPP_20–29_ fibrils (Nielsen et al., 2009; Andreasen et al., 2012). A similar fibril morphology is also evident in the hIAPP-F:Zn sample (Figure 10). This sample was prepared by adding a 1:1 ratio of Zinc to the hIAPP-F solution and then incubated for 24 h before acquiring the AFM images. The fibrils formed in this sample exhibit the same diameter as the matured helical-structured fibrils observed in both the control hIAPP (Figure 7) and hIAPP-F samples (Figure 9).

**FIGURE 11:**
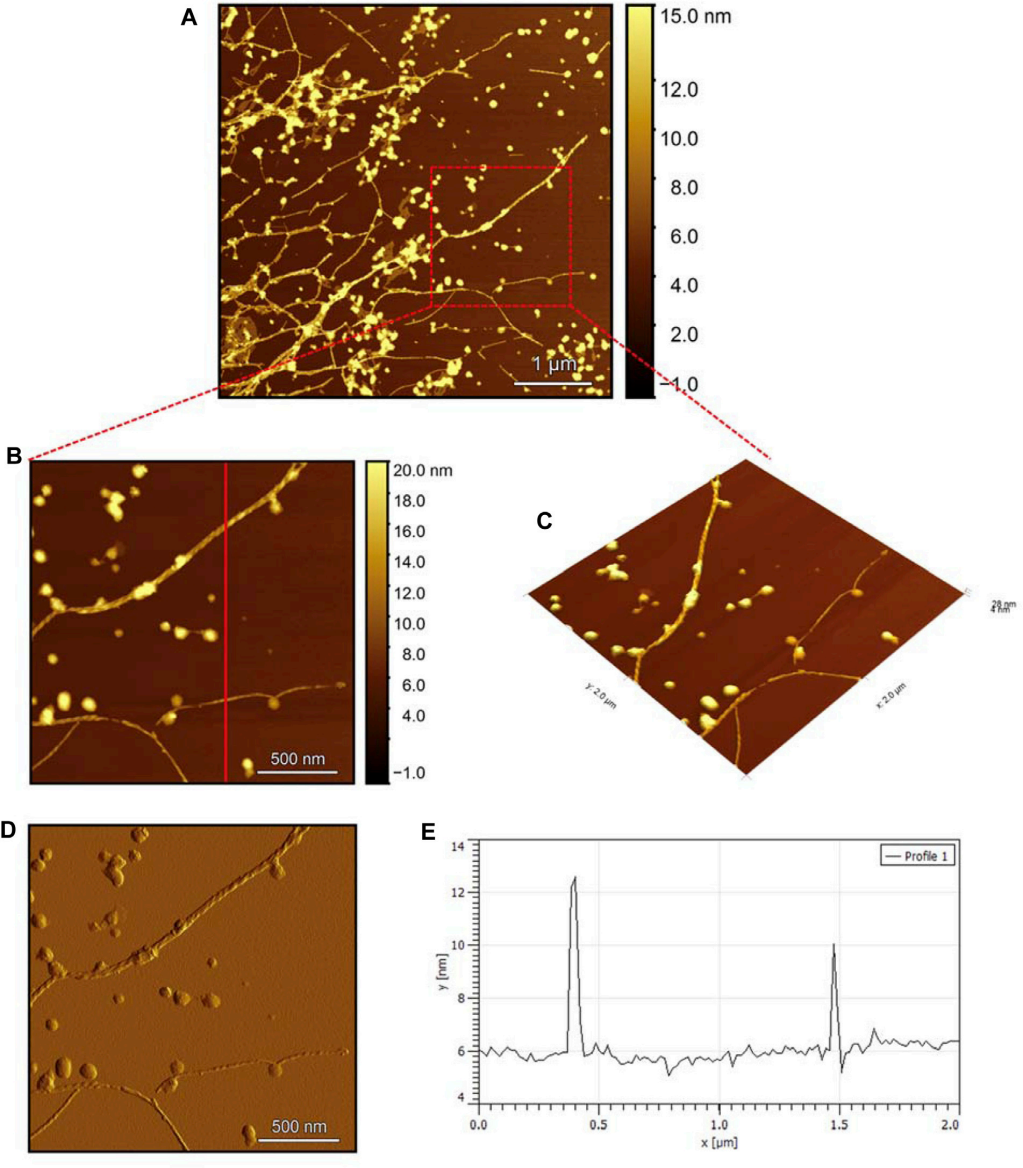
Solid-state AFM images of Pramlintide-M 200 µM sample dissolved in phosphate buffer pH 7.4. Taken after 24 h **(A)** Scan area of the formed fibrils. **(B)** 2D and **(C)** 3D views of a magnified ×22 µm area **(D)** the corresponding stiffness map of B **(E)** the corresponding profile of the diameter section analysis of **(B)**.

**FIGURE 12:**
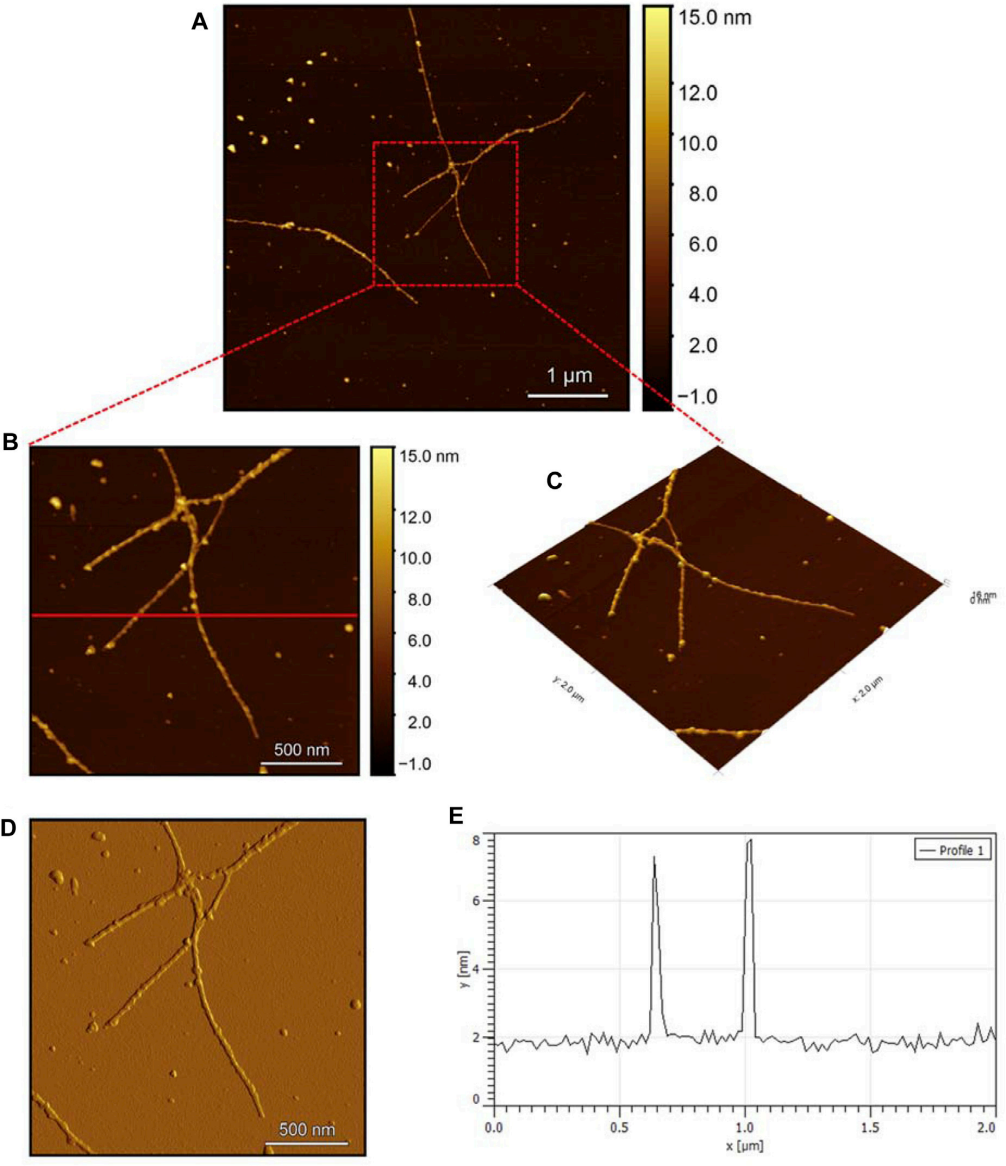
Solid-state AFM images of Pramlintide-M:Zn 200uM 1:1 ratio sample dissolved in phosphate buffer pH 7.4. Taken after 24 hrs **(A)** Scan area of the formed fibrils. **(B)** 2D and **(C)** 3D views of a magnified ×22 µm area **(D)** the corresponding stiffness map of B **(E)** the corresponding profile of the diameter section analysis of **(B)**.

Observing the Pramlintide-M sample under AFM after 24 h of incubation reveals the final-stage fibril formation process (Figure 11A). The fibril morphology is discernible in the zoomed images (Figure 11B, C), appearing as a helical structure. The spherical-like structures that are scattered throughout the sample may represent amorphous aggregates. The stiffness profile (Figure 11D) indicates that the Pramlintide-M sample has a smoother surface than the hIAPP-F sample. This difference can be distinguished by the color contrast, with brighter colors signifying roughness and darker colors indicating smoother features (Figure 11D). The diameter profile analysis shows that the fibrils exhibit the same width as the control sample, measuring 6 nm in diameter (Figure 11E). Twisted fibrils are also evident upon the addition of Zn(II) (Figure 12A). The same morphology and spherical amorphous aggregates are observed in the zoomed and 3D images (Figures 12B, C). The stiffness profile of the pramlintide-M:Zn(II) sample (Figure 12D) shows a slight variation in colors compared to the pramlintide-M sample, with pramlintide-M:Zn(II) fibrils appearing slightly brighter, indicating a rougher surface. Furthermore, the diameter of the fibrils in the pramlintide-M:Zn(II) sample matches that of the pramlintide-M sample, measuring 6 nm (Figure 12E).

Overall, this comprehensive AFM analysis explored the structural characteristics of fibril species formed by hIAPP-F and Pramlintide-M peptides after a 24-h incubation period, representing their final fibrillation stage. The results show that the morphology of the fibrils in each sample is approximately the same. The hIAPP control sample was used to make a comparison between the fibrils, showing that hIAPP exhibited varying fibril lengths with shorter worm-like fibrils. Further examination revealed two distinct fibril types–thinner ribbon-like structures and helical fibrils. The addition of Cu(II) to hIAPP produced shorter fibrils with a unique looping structure, indicating a morphological change. All samples exhibited fibrils with a helical/twisted structure, which resembles the fully mature fibrils. The hIAPP-F sample displayed elongated, twisted fibrils consistent with matured amyloid structures observed previously. Similarly, the hIAPP-F:Zn sample exhibited helical-structured fibrils akin to the control. Pramlintide samples revealed helical fibrils and spherical aggregates with smoother surfaces compared to hIAPP. Pramlintide-M:Zn(II) samples displayed twisted fibrils and minor variations in stiffness. The diameter of fibrils across samples consistently measured 6 nm.

### 3.4 Assessing peptide cytotoxicity: impact of metal ions on cell viability and aggregation

The cytotoxicity test was conducted to assess the potential toxicity of each peptide, both individually and in combination with the metal ions Cu(II) and Zn(II), on HeLa cells. Controls of Cu(NO_3_)_2_ and ZnCl_2_ were used (Supplementary Figure S12) in order to confirm that their concentrations were not toxic to the cells. As the full-length hIAPP peptide is known to form toxic aggregates within cells, it served as a positive control for comparison with the effects of the shorter peptide fragments. Initially, the test was performed on HeLa cells by adding each peptide alone. Subsequently, the peptides that exhibited the most significant effects were selected for further testing with different metal ions.

**FIGURE 13:**
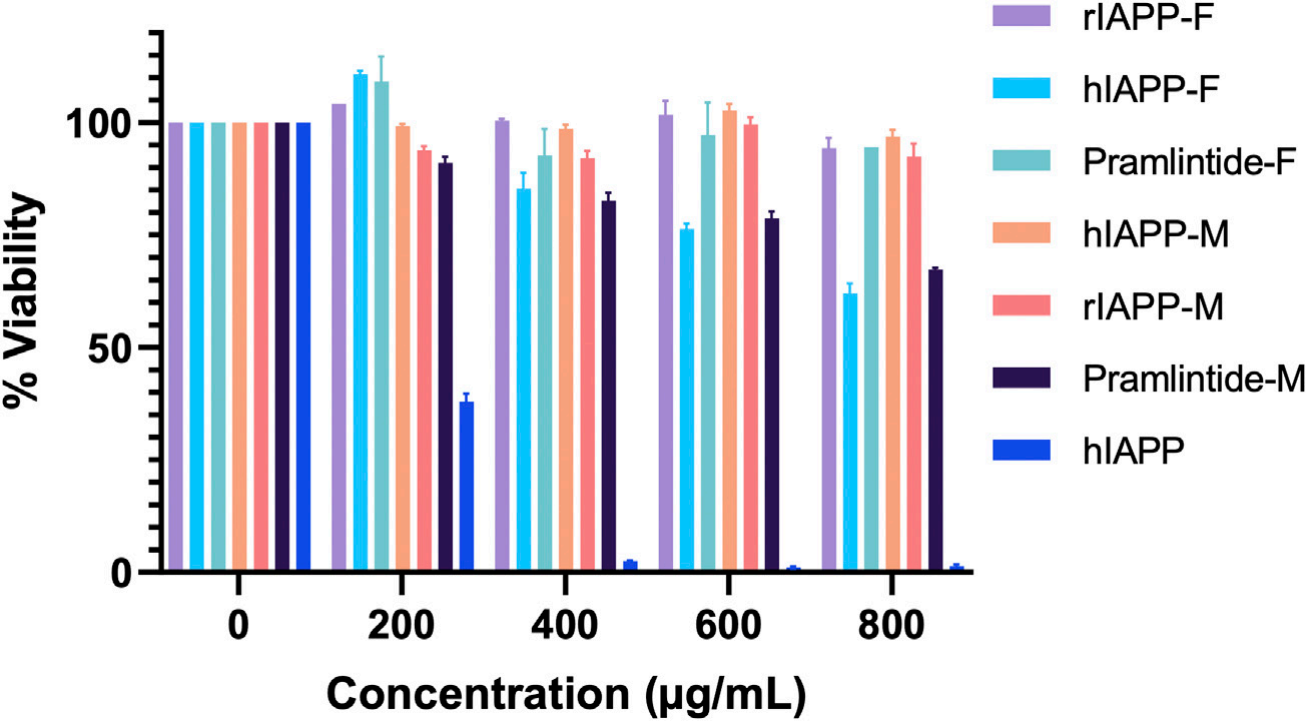
The cytotoxic effect of each peptide at different concentrations (0–800 μg/mL) on the HeLa cells. Error bars are based on SEM (n = 3).

The results for the non-aggregating short peptides, namely, rIAPP-F, Pramlintide-F, hIAPP-M, and rIAPP-M, demonstrated no adverse effects on cell viability, which aligns with our ThT assay findings (Figure 13). On the other hand, the hIAPP peptide (used as the positive control) exhibited the highest level of toxicity to the cells, as anticipated. Its effects become evident even at lower concentrations, starting at 200 μg/mL and reaching maximum toxicity at 400 μg/mL, resulting in 0% cell viability (Figure 13). Furthermore, Pramlintide-M decreased the viability of cells, with its effect becoming more pronounced as the concentration increased, eventually resulting in a 70% cell viability rate at the highest concentration tested, 800 μg/mL. Likewise, hIAPP-F exhibited a similar effect on cell viability, with a maximum concentration of 800 μg/mL resulting in 60% cell viability. Based on these findings, both peptides were selected for further assessment of their toxicity, particularly in conjunction with the addition of Cu(II) and Zn(II) ions.

**FIGURE 14:**
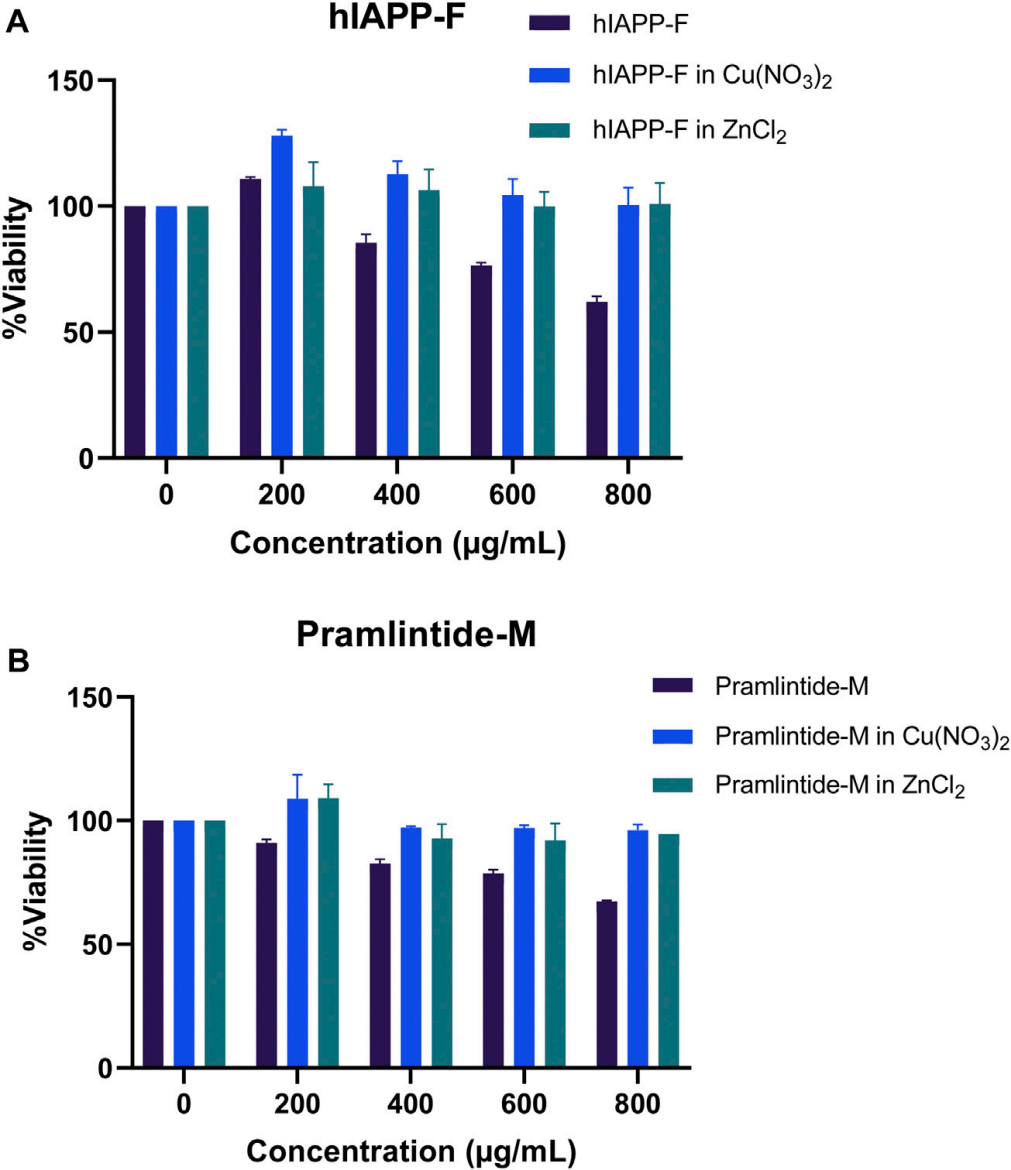
The cytotoxic effect of selected peptides, Pramlintide-M (dark blue), Pramlintide-M in Cu(NO_3_)_2_ (blue) and Pramlintide-M in ZnCl_2_ (green) at different concentrations (0–800 μg/mL) on HeLa cells with and without the addition of Cu(II) and Zn(II). Error bars are based on SEM (*n* = 3).

Both the hIAPP-F and Pramlintide-M peptide-Cu(II) complexes demonstrated high cell viability across the various concentrations tested (Figure 14). These results support our earlier findings from the ThT assay and AFM, which indicated a low rate of fibril formation and aggregation in the presence of Cu(II) in the solution. Surprisingly, they also exhibited a high cell proliferation rate with the addition of Zn(II) to the solution (Figure 14). Based on our observation, we can postulate that Zn(II) ions may possess specific protective properties against aggregation within the cells (*in vivo* experiment), which can be manifested by the elevated cell viability observed when peptide-Zn(II) ions complexes were introduced. In contrast, during *in vitro* experiments, Zn(II) ions, when combined with the peptides hIAPP-F and Pramlintide-M, appeared to either promote aggregation or yield the same aggregation rate, respectively, when compared to the peptides alone. This suggests the presence of other interactions within the cellular environment that affect the aggregation of the peptide fragments in the presence of Zn(II). It appears that the intricate environment of the cell, where numerous interactions take place, modulates the interaction between Zn(II) and the short peptides hIAPP-F and Pramlintide-M. This modulation subsequently reduces the cytotoxicity of the peptide-Zn(II) complexes within the cell, in contrast to *in vitro* conditions where these additional interactions are absent.

## 4 Conclusion

This study aimed to investigate the influence of amino acid substitutions in the central region of amylin and its analogs on peptide aggregation. The ThT assay was employed to evaluate the aggregation tendencies of these peptides both with and without the addition of specific metal ions. Afterward, AFM was utilized to examine the morphology of the fibril species formed by peptides that exhibited aggregation behavior in the ThT assay, which were hIAPP-F and Pramlintide-M. As anticipated, the hIAPP-F showed increased fluorescence intensity, indicating aggregation. The sudden addition of Zn(II) enhanced aggregation, acting as a promoter, whereas the application of Cu(II) significantly decreased fluorescence intensity, acting as an inhibitor. Pramlintide-M and Pramlintide-M:Zn(II) showed a slight increase in fluorescence intensity. The peptide alone aggregated after 7 h, while the Zn(II)-containing solution exhibited delayed aggregation onset after 12 h. Subsequently, both reached saturation after 14 h. This increase in fluorescence was attributed to the mutation of the last two prolines to serins, demonstrating the role of prolines in inhibiting β-sheet formation and aggregation. Other Peptides, namely, rIAPP-F, pramlintide-F, hIAPP-M, and rIAPP-M did not exhibit increased fluorescence intensity, and both Cu(II) and Zn(II) had no influence on their aggregation rates. The presence of proline residues in their primary sequences likely contributed to this lack of aggregation. Interestingly, hIAPP-M, which had only the last two serins mutated to prolines, did not exhibit aggregation, suggesting that serines play a crucial role in β-sheet formation due to their -OH group’s involvement in creating hydrogen bond networks. Moreover, one may say that proline residues can expose their full potential for being a beta-sheet breaker when they are placed within the amino acid sequences as well as at their end.

NMR spectroscopy confirmed the aggregation of peptides hIAPP-F and pramlintide-M. It showed that the aggregation process of hIAPP-F is accelerated in the presence of Zn(II). This aligns with the results obtained for these systems by ThT and AFM. Lastly, the cytotoxicity assay conducted on the HeLa cell line yielded several significant findings. Specifically, rIAPP-F, Pramlintide-F, hIAPP-M, and rIAPP-M showed no significant effects on cell viability, while hIAPP-F and Pramlintide-M demonstrated a decrease in cell viability. To investigate these effects further, we selected hIAPP-F and Pramlintide-M for evaluation in conjunction with Cu(II) and Zn(II). Notably, peptide-Cu(II) complexes of hIAPP-F and Pramlintide-M exhibited high cell viability across various concentrations, supporting our earlier ThT assay and AFM results. However, a surprising observation emerged when Zn(II) was introduced to the solution, as both complexes led to an increased proliferation rate of cells. This intriguing result suggests that Zn(II) ions may possess protective properties against aggregation within cells, particularly in *in vivo* experiments, where elevated cell viability was observed upon the addition of peptide-Zn(II) complexes.

To complete, these *in vitro* studies have revealed the assertive amyloidogenic nature of human Islet Amyloid Polypeptide (hIAPP), with the region from 20 to 29 playing a pivotal role in dictating its amyloid-forming ability. This observation gains further support from investigations involving ten residue peptides derived from the region 20 to 29 of hIAPP. Interestingly, not all mammals exhibit the formation of islet amyloid, notably mice and rats. A comparative analysis of the rat/mouse sequence with that of hIAPP supports the hypothesis that the 20–29 segment complexly controls the capability to form amyloid. Notably, human and rat IAPP (rIAPP) sequences differ at six positions, with five of these disparities concentrated between residues 23–29. A remarkable distinction is the presence of three Proline residues at positions 25, 28, and 29 in the rat sequence, while the human analog lacks them (Rivera et al., 2009). Given Proline’s disruption of β-sheet stability and its unfavorable energetic profile, the inability of rat IAPP to form amyloid has been attributed to these Proline substitutions.

## Data Availability

The original contributions presented in the study are included in the article/Supplementary Material, further inquiries can be directed to the corresponding author.
