# Supplementary material for: Exploring the central region of amylin and its analogs aggregation: the influence of metal ions and residue substitutions
Source: Front Chem. 2024 Jul 8;12:1419019. doi: 10.3389/fchem.2024.1419019 (PMC11272978; doi:10.3389/fchem.2024.1419019)
Supplement: Supplementary file 1 [file DataSheet2.docx]

Table S1: ^1^H NMR Chemical Shift Assignments of mutants 1, 5, 7 and 9 (δ in ppm Relative to TMS)

| **Peptide** | **a.a.** | **HN** | **H**α | **H**β | **H**ɣ | **H**δ |
| --- | --- | --- | --- | --- | --- | --- |
| **rIAPP-F** | S1 | n.a. | n.a. | n.a. | n.a. | n.a. |
|  | N2 | n.a. | 4.789 | 2.801 2.881 | – | – |
|  | N3 | n.a. | 4.734 | 2.768 2.857 | – | – |
|  | L4 | n.a. | 4.414 | 1.652 1.698 | 1.656 | 0.888 0.951 |
|  | G5 | 8.122 | 4.054 4.139 | – | – | – |
|  | P6 | – | 4.482 | 1.917 2.269 | 2.027 | 3.643 3.639 |
|  | V7 | 8.182 | 4.091 | 2.048 | 0.935 0.968 | – |
|  | L8 | 8.265 | 4.712 | 1.579 1.614 | 1.670 | 0.962 0.962 |
|  | P9 | – | 4.691 | 2.010 2.356 | 2.108 | 3.658 3.891 |
|  | P10 | – | 4.270 | 1.921 2.237 | 2.013 | 3.647 3.791 |
| **Pramlintide-F** | S1 | n.a. | n.a. | n.a. | n.a. | n.a. |
|  | N2 | n.a. | 4.763 | 2.734 2.774 | – | – |
|  | N3 | n.a. | 4.727 | 2.761 2.779 | – | – |
|  | F4 | n.a. | 4.749 | 3.080 3.298 | – | – |
|  | G5 | 8.117 | 4.132 4.132 | – | – | – |
|  | P6 | – | 4.506 | 1.953 2.303 | 2.060 | 3.657 3.666 |
|  | I7 | n.a. | 4.197 | 1.894 | 0.935 \| 1.255 1.565 | 0.927 |
|  | L8 | 8.238 | 4.757 | 1.616 1.645 | 1.698 | 0.962 0.985 |
|  | P9 | – | 4.721 | 2.045 2.379 | 2.140 | 3.685 3.913 |
|  | P10 | – | 4.304 | 1.958 2.273 | 2.050 | 3.679 3.824 |
| **hIAPP-M** | S1 | n.a. | n.a. | n.a. | n.a. | n.a. |
|  | N2 | n.a. | 4.772 | 2.763 2.808 | – | – |
|  | N3 | n.a. | 4.739 | 2.707 2.814 | – | – |
|  | F4 | n.a. | 4.665 | 3.121 3.288 | – | – |
|  | G5 | 8.294 | 3.929 3.930 | – | – | – |
|  | A6 | n.a. | 4.406 | 1.432 | – | – |
|  | I7 | 9.074 | 4.228 | 1.917 | 0.944 \| 1.252 1.549 | 0.933 |
|  | L8 | 8.259 | 4.754 | 1.626 1.649 | 1.708 | 0.976 0.997 |
|  | P9 | – | 4.727 | 2.057 2.391 | 2.154 | 3.695 3.924 |
|  | P10 | – | 4.318 | 1.972 2.281 | 2.058 | 3.689 3.838 |
| **rIAPP-M** | S1 | n.a. | n.a. | n.a. | n.a. | n.a. |
|  | N2 | n.a. | 4.807 | 2.824 2.899 | – | – |
|  | N3 | n.a. | 4.742 | 2.788 2.877 | – | – |
|  | L4 | n.a. | 4.427 | 1.666 1.712 | 1.661 | 0.905 0.958 |
|  | G5 | 8.130 | 4.073 4.144 | – | – | – |
|  | P6 | – | 4.485 | 1.930 2.282 | 2.042 | 3.646 3.670 |
|  | I7 | n.a. | 4.171 | 1.871 | 0.909 \| 1.235 1.537 | 0.906 |
|  | L8 | n.a. | 4.736 | 1.602 1.634 | 1.679 | 0.937 0.972 |
|  | P9 | – | 4.700 | 2.027 2.368 | 2.130 | 3.673 3.904 |
|  | P10 | – | 4.286 | 1.939 2.257 | 2.028 | 3.662 3.809 |

Table S2: ^13^C NMR Chemical Shift Assignments of mutants 1, 5, 7 and 9 (δ in ppm Relative to TMS)

| **Peptide** | **a.a.** | **C**α | **C**β | **C**ɣ | **C**δ |
| --- | --- | --- | --- | --- | --- |
| **rIAPP-F** | S1 | n.a. | n.a. | n.a. | n.a. |
|  | N2 | 52.265 | 38.017 | – | – |
|  | N3 | 52.45 | 37.915 | – | – |
|  | L4 | 54.347 | 41.522 | 26.140 | 22.355 24.173 |
|  | G5 | 43.641 | – | – | – |
|  | P6 | 62.122 | 31.197 | 26.365 | 48.941 |
|  | V7 | 61.501 | 31.703 | 20.285 19.787 | – |
|  | L8 | 51.667 | 40.901 | 26.128 | 22.431 24.173 |
|  | P9 | 60.473 | 29.741 | 26.452 | 49.684 |
|  | P10 | 63.714 | 31.161 | 26.295 | 49.24 |
| **Pramlintide-F** | S1 | n.a. | n.a. | n.a. | n.a. |
|  | N2 | 52.162 | 37.989 | – | – |
|  | N3 | 52.277 | 37.804 | – | – |
|  | F4 | n.a. | 38.544 | – | – |
|  | G5 | 41.906 | – | – | – |
|  | P6 | 60.387 | 29.494 | 24.597 | 47.211 |
|  | I7 | 58.381 | 35.919 | 14.908 24.668 | 10.058 |
|  | L8 | 49.799 | 39.279 | 24.402 | 20.725 22.473 |
|  | P9 | 58.726 | 28.021 | 24.681 | 47.978 |
|  | P10 | 62.130 | 29.486 | 24.606 | 47.492 |
| **hIAPP-M** | S1 | n.a. | n.a. | n.a. | n.a. |
|  | N2 | 53.021 | 38.868 | – | – |
|  | N3 | 53.092 | 38.627 | – | – |
|  | F4 | n.a. | 39.06 | – | – |
|  | G5 | 45.117 | – | – | – |
|  | A6 | 52.147 | 19.448 | – | – |
|  | I7 | 60.842 | 38.638 | 17.471 27.125 | 12.654 |
|  | L8 | 52.432 | 41.862 | 26.992 | 23.388 25.050 |
|  | P9 | 61.328 | 30.614 | 27.228 | 50.569 |
|  | P10 | 64.722 | 32.073 | 27.226 | 50.085 |
| **rIAPP-M** | S1 | n.a. | n.a. | n.a. | n.a. |
|  | N2 | 52.337 | 38.047 | – | – |
|  | N3 | 52.578 | 37.838 | – | – |
|  | L4 | 54.341 | 41.593 | 26.154 | 22.392 24.161 |
|  | G5 | 43.64 | – | – | – |
|  | P6 | 62.172 | 31.247 | 26.386 | 48.956 |
|  | I7 | 60.106 | 37.653 | 16.675 26.446 | 11.839 |
|  | L8 | 51.718 | 41.018 | 26.197 | 22.506 24.220 |
|  | P9 | 60.541 | 29.867 | 26.499 | 49.768 |
|  | P10 | 63.806 | 31.238 | 26.386 | 49.316 |
