# Supplementary material for: Exploring the central region of amylin and its analogs aggregation: the influence of metal ions and residue substitutions
Source: Front Chem. 2024 Jul 8;12:1419019. doi: 10.3389/fchem.2024.1419019 (PMC11272978; doi:10.3389/fchem.2024.1419019)
Supplement: Supplementary file 2 [file DataSheet1.docx]

SUPPLEMENTARY MATERIAL

**Exploring the Central region of Amylin and Analog Aggregation: The Influence of Metal Ions and Residue Substitutions**

*Mawadda Alghrably^1^, Giulia Bennici^1^Gabriela Szczupaj, Batoul Maatouk, Somayah Qutub, Noura Alasmael, Kousik Chandra, Michal Nowakowski, Abdul-Hamid Emwas, Niveen Khashab, , Mariusz Jaremko^1^*

# Reagents:

All reagents (copper(II) nitrate, zinc(II) chloride, Thioflavin T (ThT), 50 mM HEPES buffer) were purchased from Fisher Scientific. The reagents were of the highest grade commercially available and were used without further purification.

# Thioflavin T

Controls of Cu(NO_3_)_2_ and ZnCl_2_ were used at a concentration of 40 μM (Figure S1) to ensure that they did not interfere with the aggregation results. hIAPP-F, pramlintide-F, hIAPP-M, and rIAPP-M exhibited no increase in fluorescence intensity (Figure S2). Furthermore, the aggregation rate of these four peptides was unaffected by the presence of either Cu(II) or Zn(II) ions (Figure S3). This phenomenon can be attributed to the presence of proline residues within the primary sequence of each peptide.


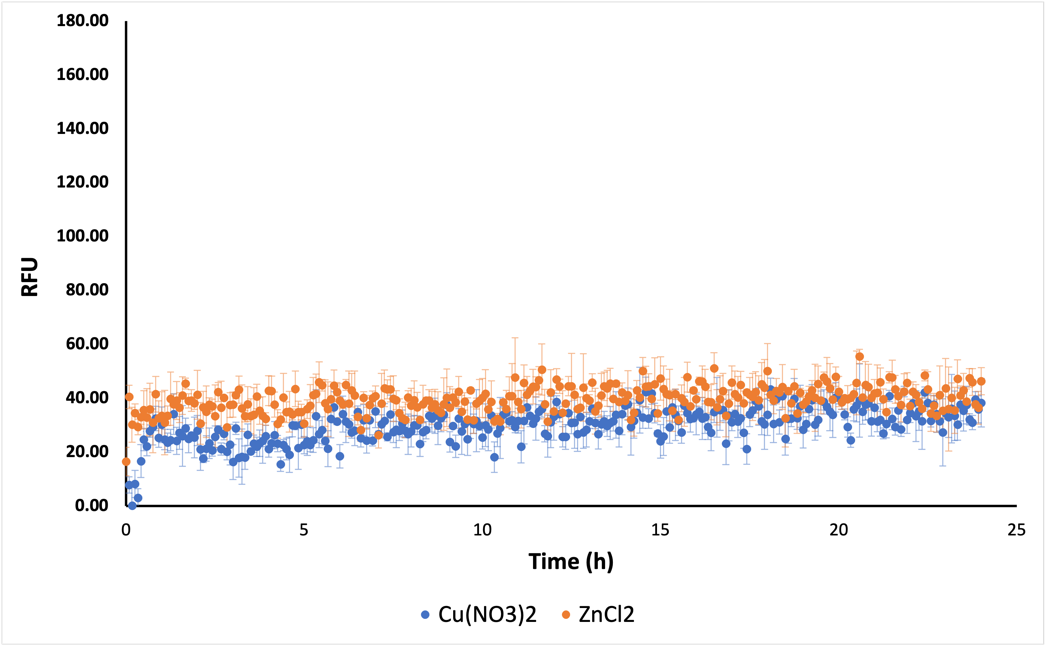


**Figure S1**. Control samples of Cu(NO_3_)_2_ and ZnCl_2_ (40 μM) in the presence of ThT (20 μM) fluorescence and 50 mM phosphate buffer pH 7.4 at 25°C

**
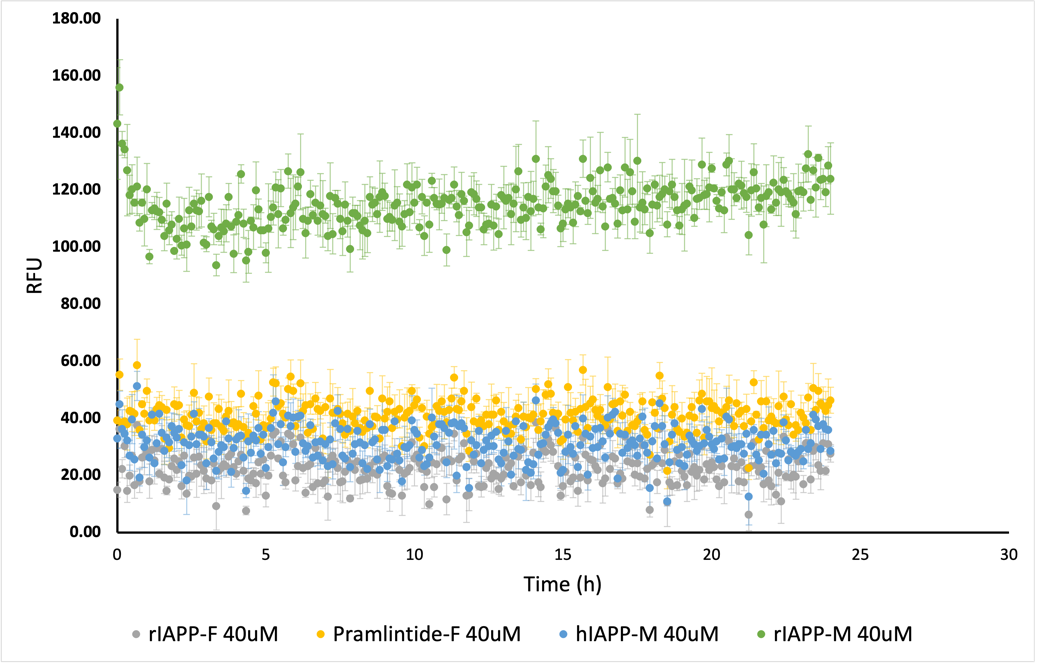
**

**Figure S2**. rIAPP-F, Pramlintide-F, hIAPP-M and rIAPP-M did not show aggregation in a phosphate-buffered solution. Amylin peptides (40μM) were subjected to aggregation at 25°C in the presence of 20μM ThT and 50mM phosphate buffer pH 7.4.


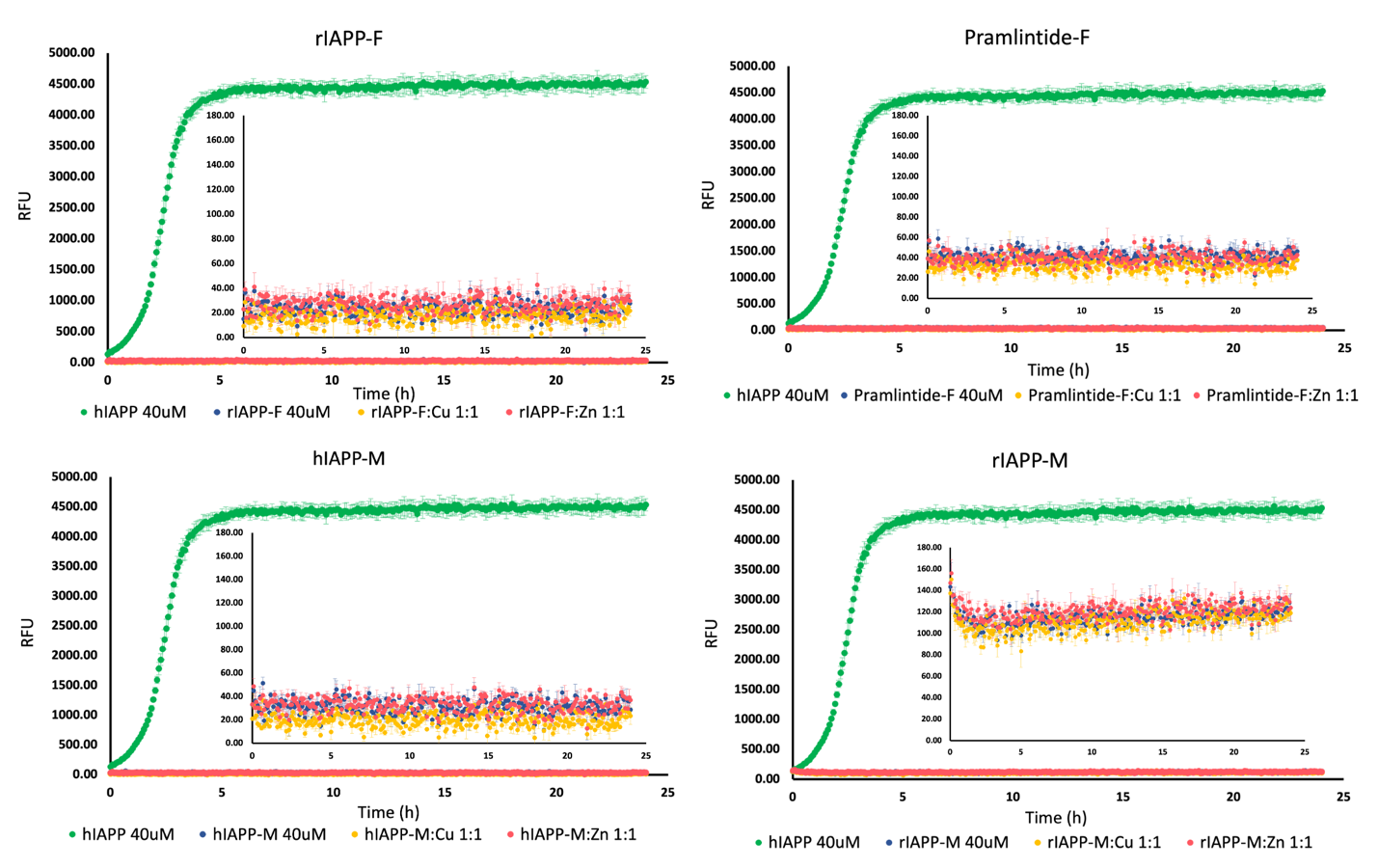


**Figure S3**. Effects of Cu(II) and Zn(II) on rIAPP-F, Pramlintide-F, hIAPP-M and rIAPP-M aggregation in a phosphate-buffered solution. Amylin peptides (40 μM) were subjected to aggregation at 25°C in the presence of 20 μM ThT and 50 mM phosphate buffer pH 7.4.

**3 NMR**

To investigate the influence of Zn(II) addition on peptide hIAPP-F structure, one-dimensional NMR spectra were overlaid (Figure S4). This analysis showed that the addition of Zn (II) did not induce any changes in the spectrum. Furthermore, for the samples containing hIAPP-F, pramlintide-M, and hIAPP-F in the presence of Zn(II) we acquired ^1^H NMR spectra immediately after sample preparation and at 4-hour intervals over a period of 44 hours. Superimposed spectra allowed us to observe signal intensity detoration which we associate with hIAPP-F aggregation whether separated or in the presence of metal ion (Figure S5).

For peptides: rIAPP-F, pramlintide-F, hIAPP-M and rIAPP-M, the assignment was transferred from two-dimensional to one-dimensional spectra (Figures S6, S7, S8, S9). Subsequently, based on the transferred assignment, the assignment of ^1^H NMR spectra of the peptides prone to aggregation was done(Figures S10, S11).

**F**
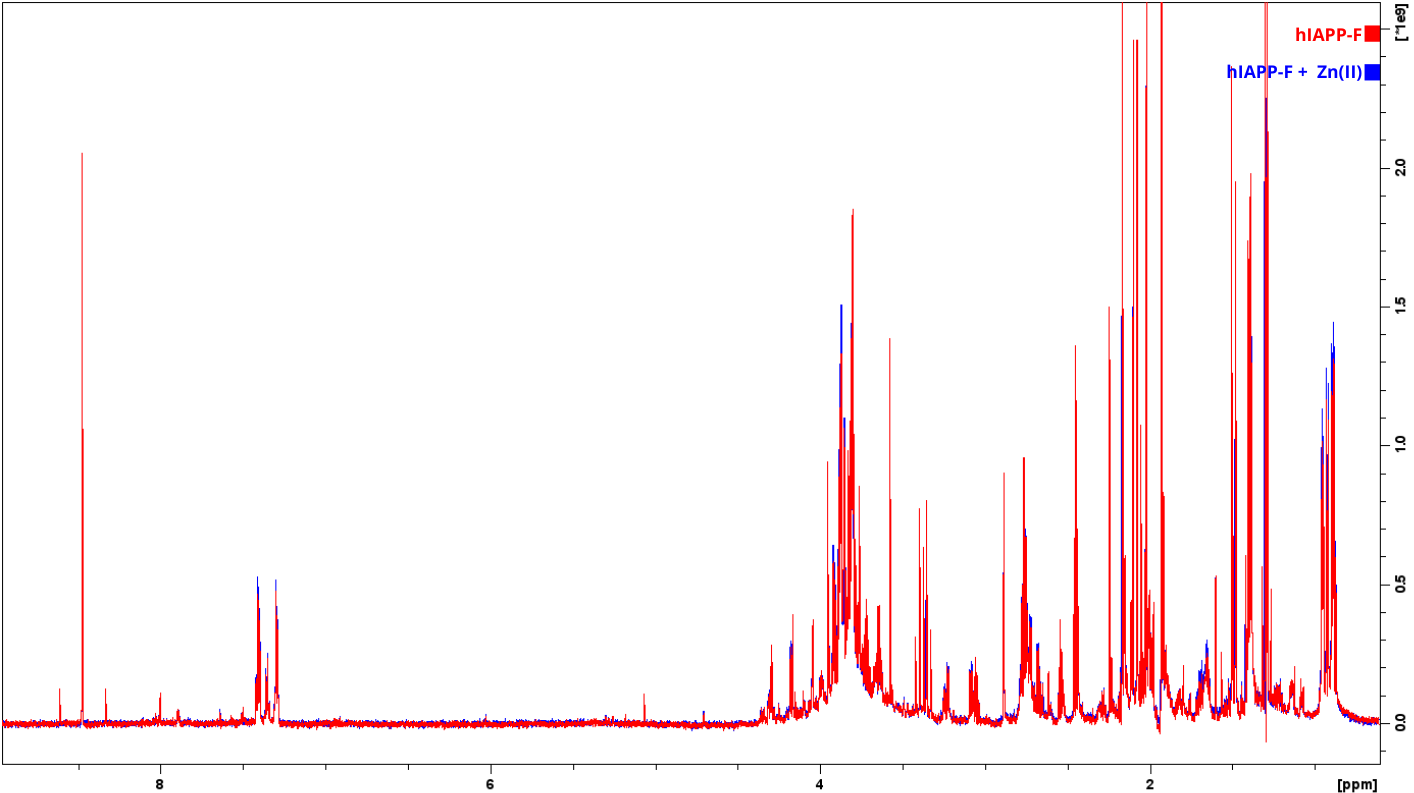
**igure S4**. Superimposed 1D ^1^H NMR spectra of peptide hIAPP-F individually and in the presence of Zn(II).


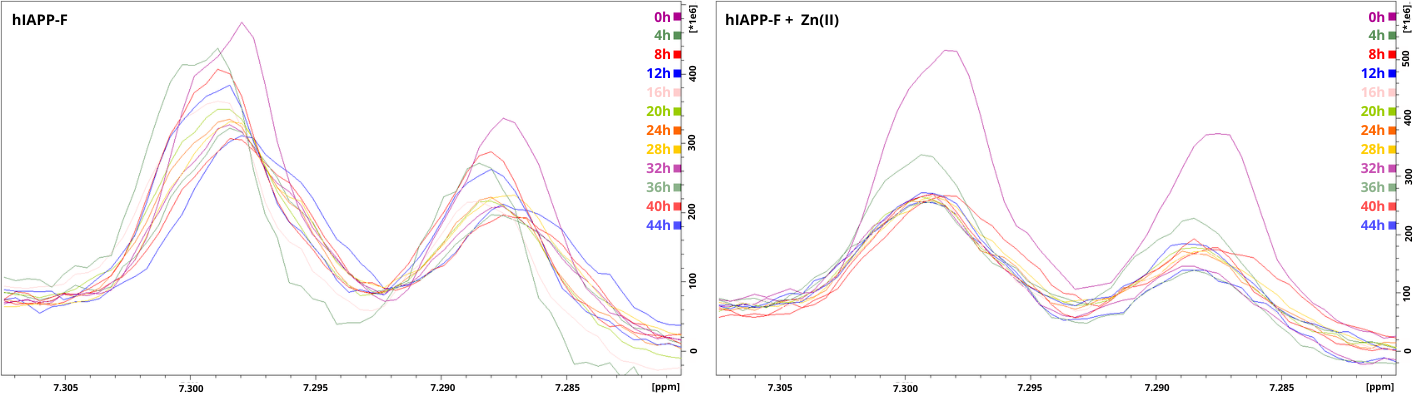

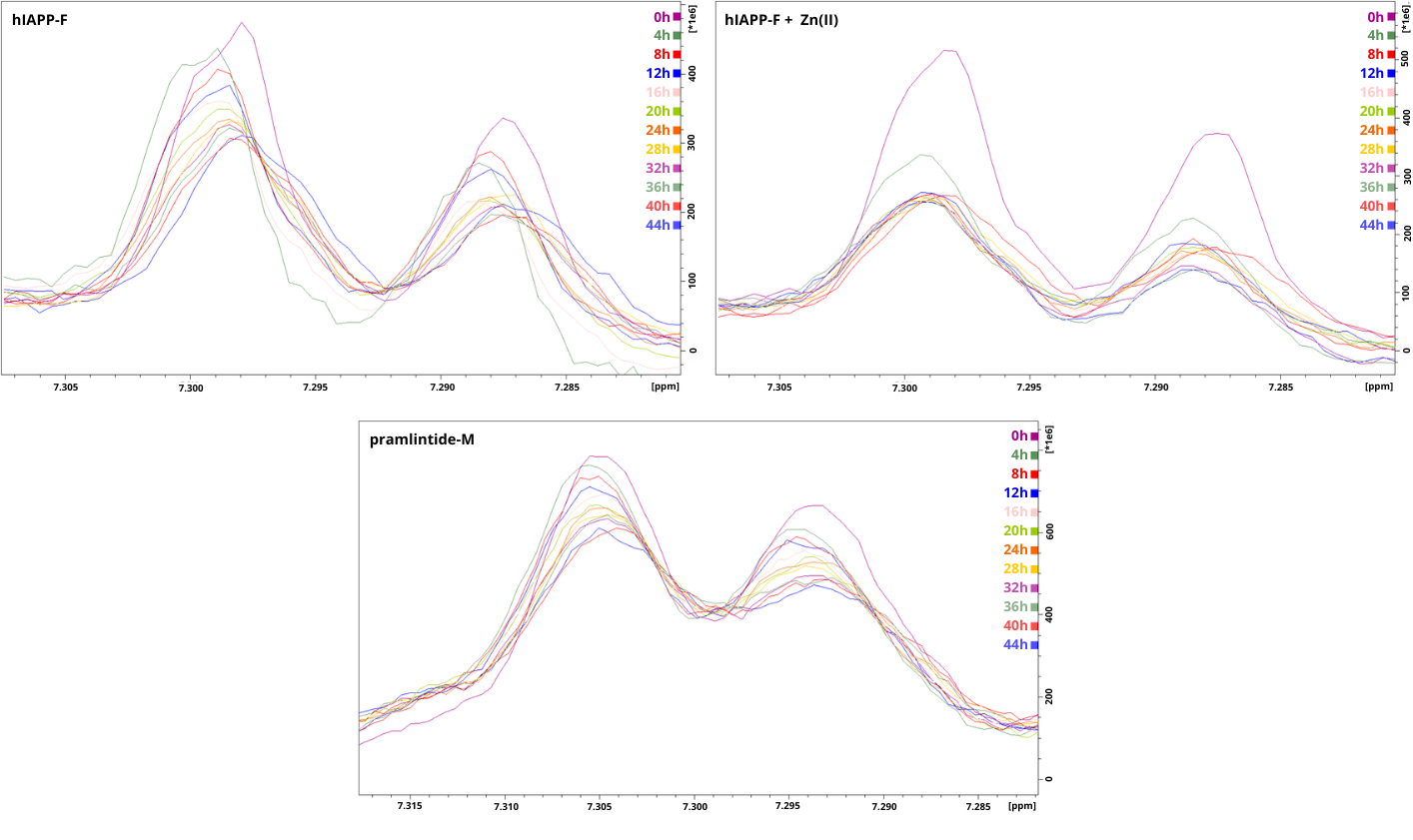


**Figure S5.** Superimposed fragments of 1D experiments for peptides hIAPP-F and pramlintide recorded individually and hIAPP-F in the presence of Zn (II) over 44 hours. Selected fragments highlight one of the aromatic signals for residue F4 and its time-dependent decrease.


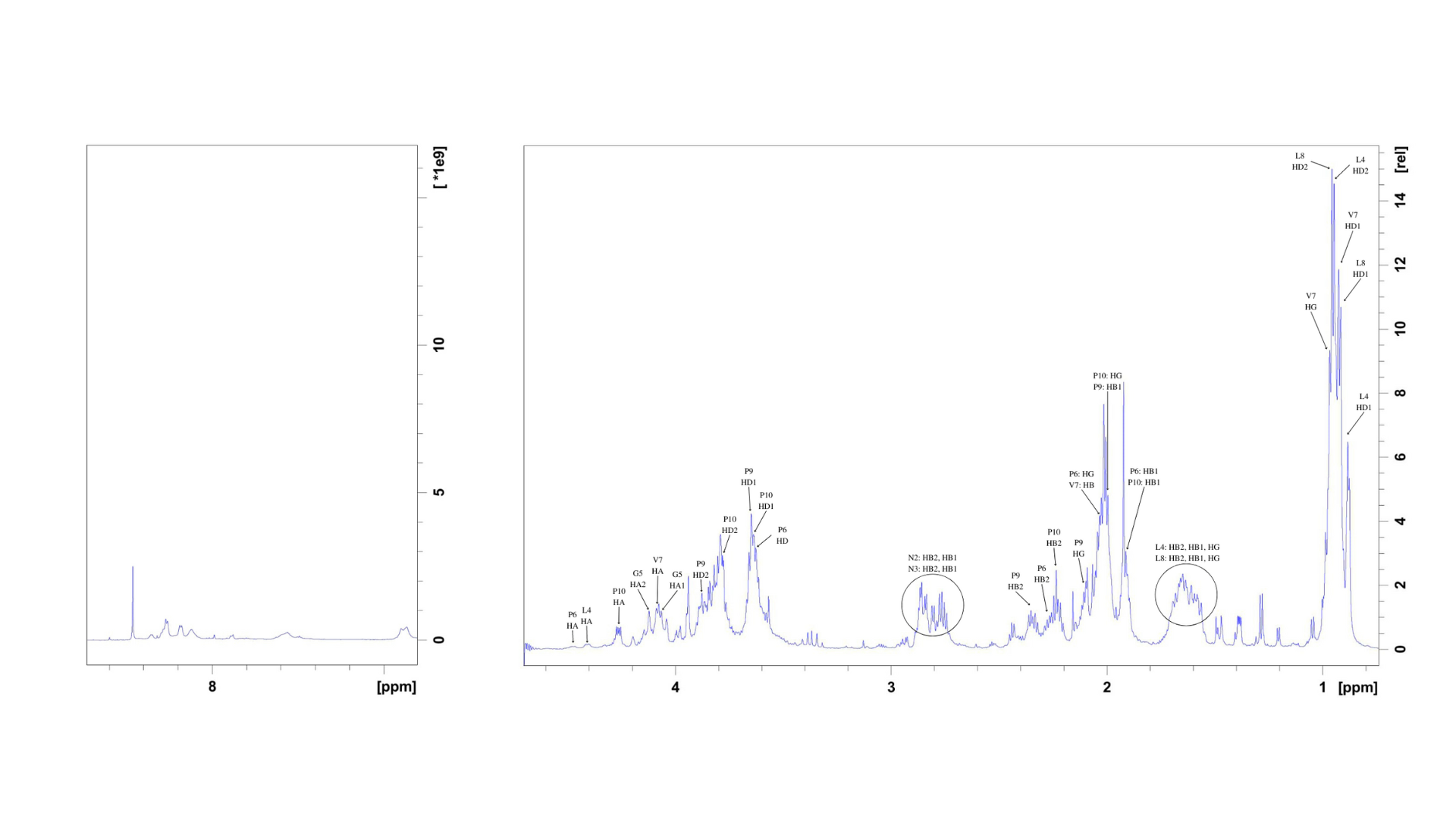


**Figure S6.** Assigned ^1^H NMR spectrum of the peptide rIAPP-F. The shown fragments present the regions of spectrum where signals of the peptide can be find.


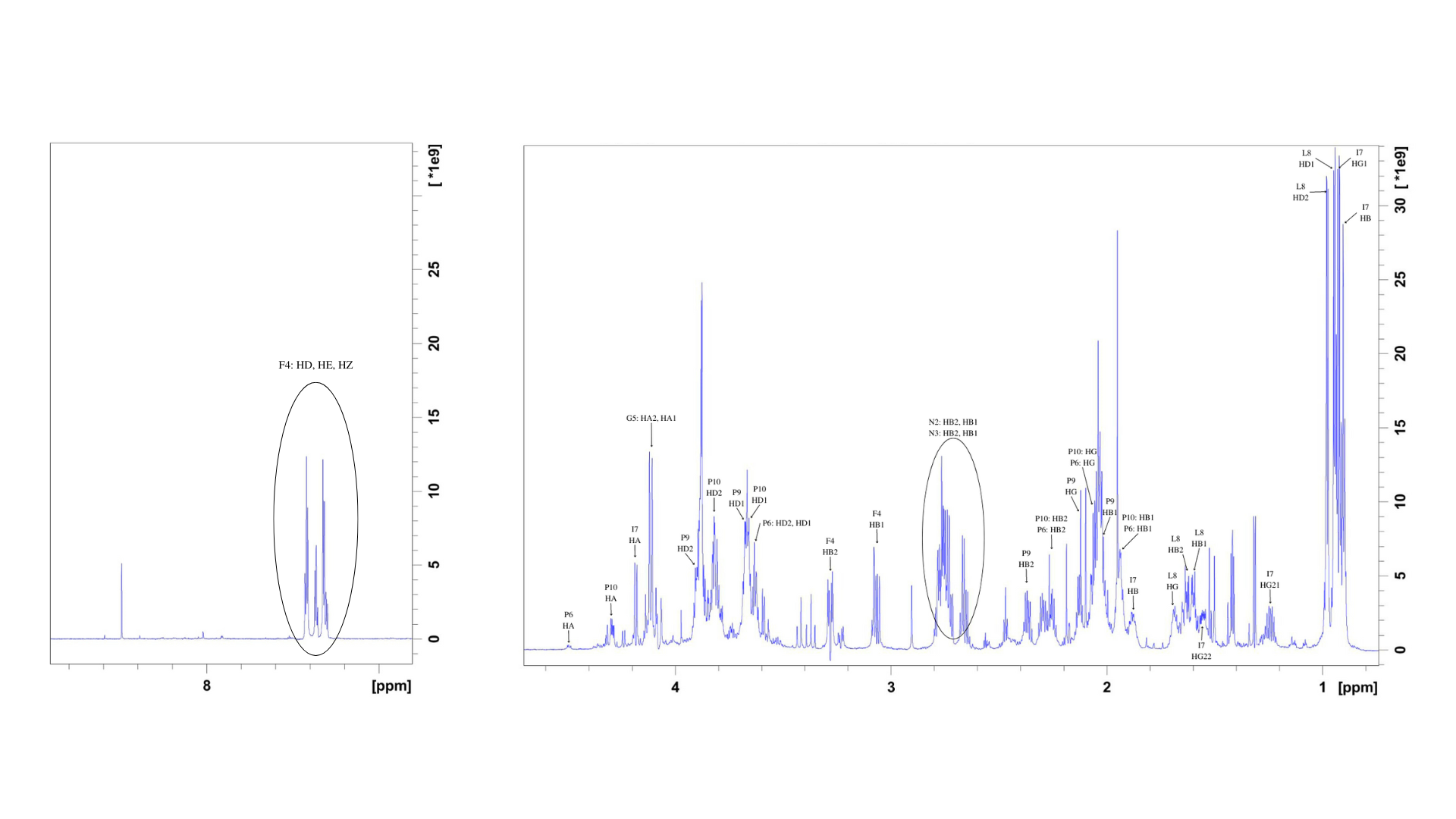


**Figure S7.** Assigned ^1^H NMR spectrum of the peptide pramlintide-F. The shown fragments present the regions of spectrum where signals of the peptide can be find.


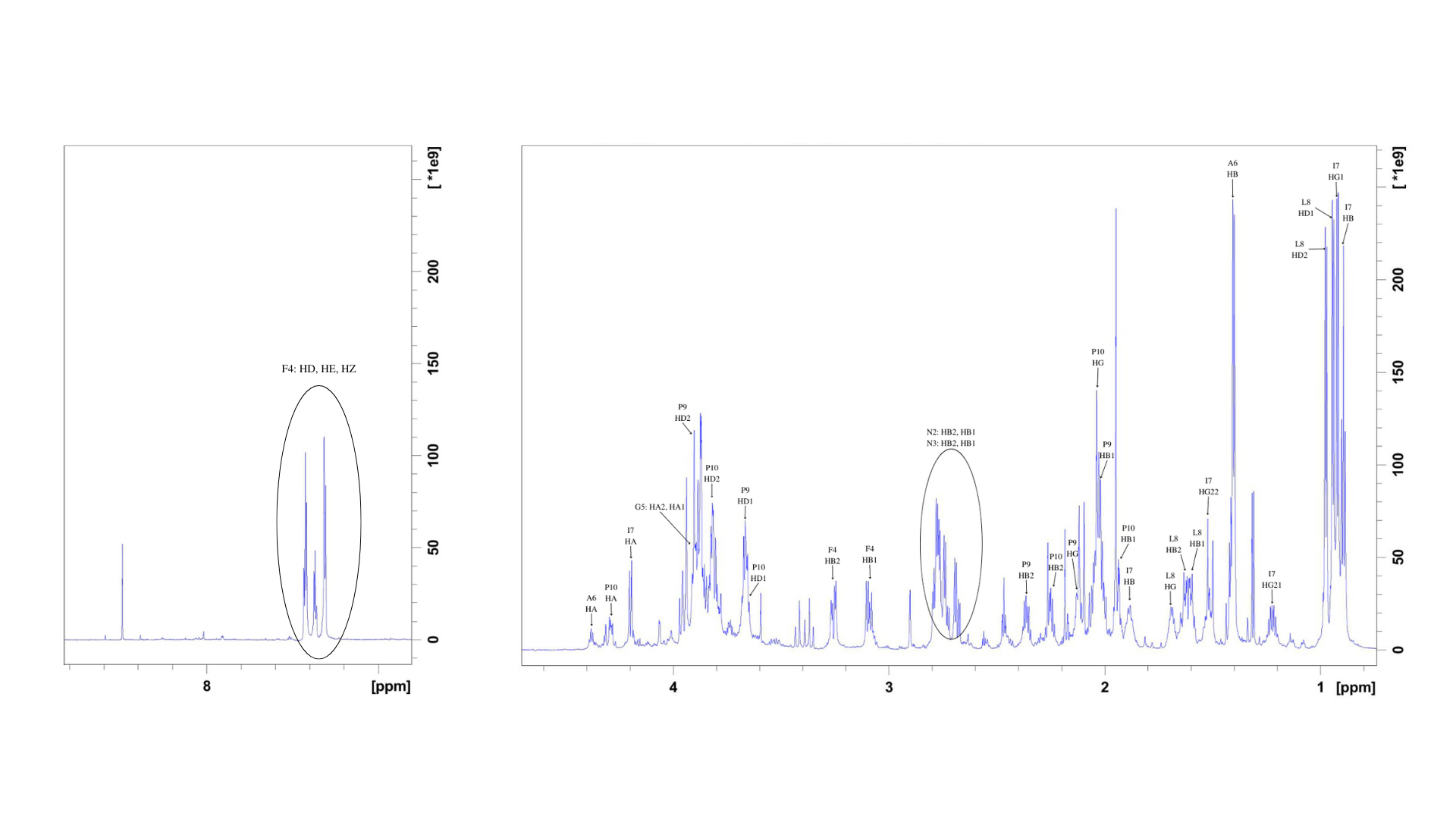


**Figure S8.** Assigned ^1^H NMR spectrum of the peptide hIAPP-M. The shown fragments present the regions of spectrum where signals of the peptide can be find.


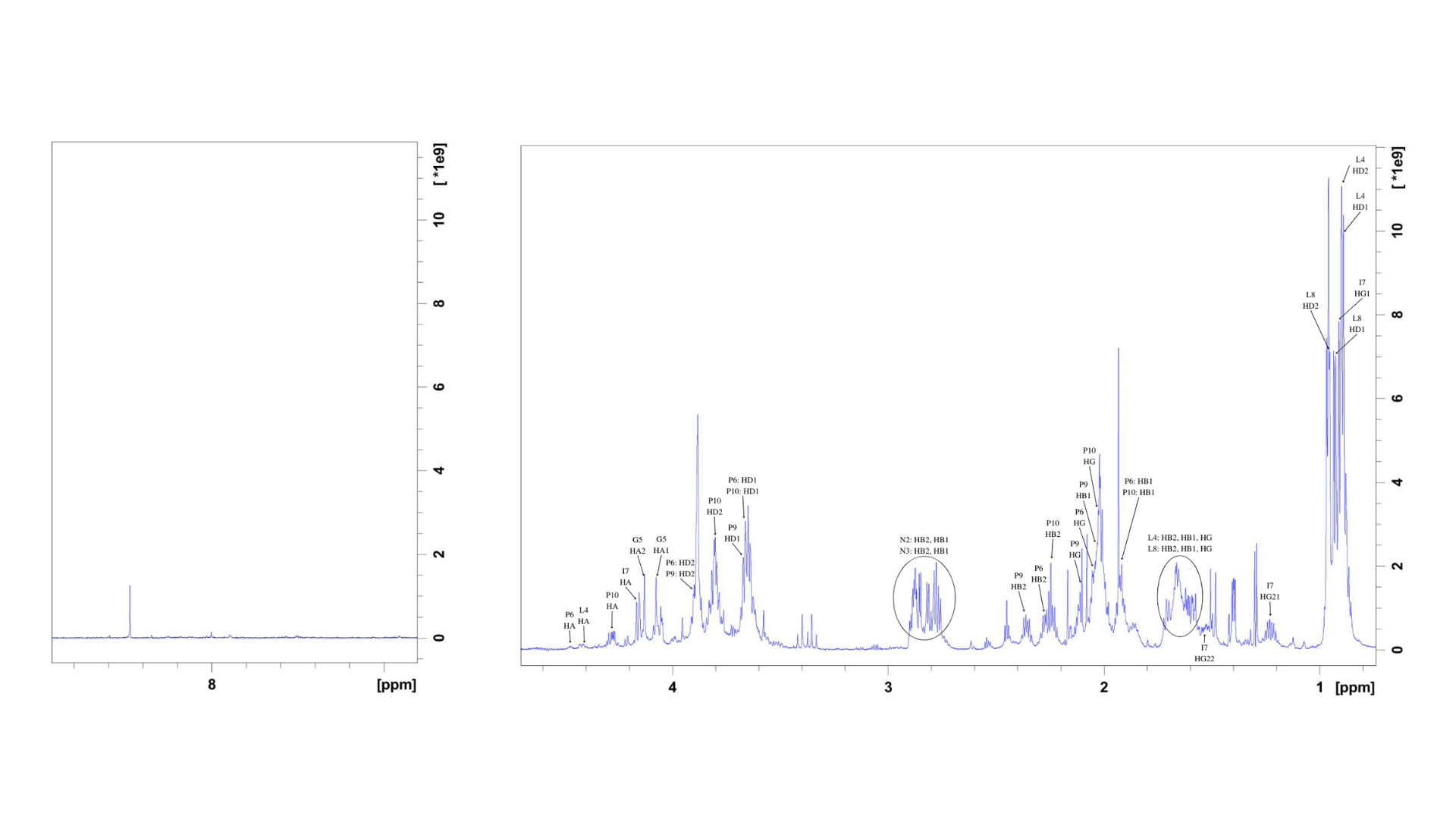
**Figure S9.** Assigned ^1^H NMR spectrum of the peptide rIAPP-M. The shown fragments present the regions of spectrum where signals of the peptide can be find.


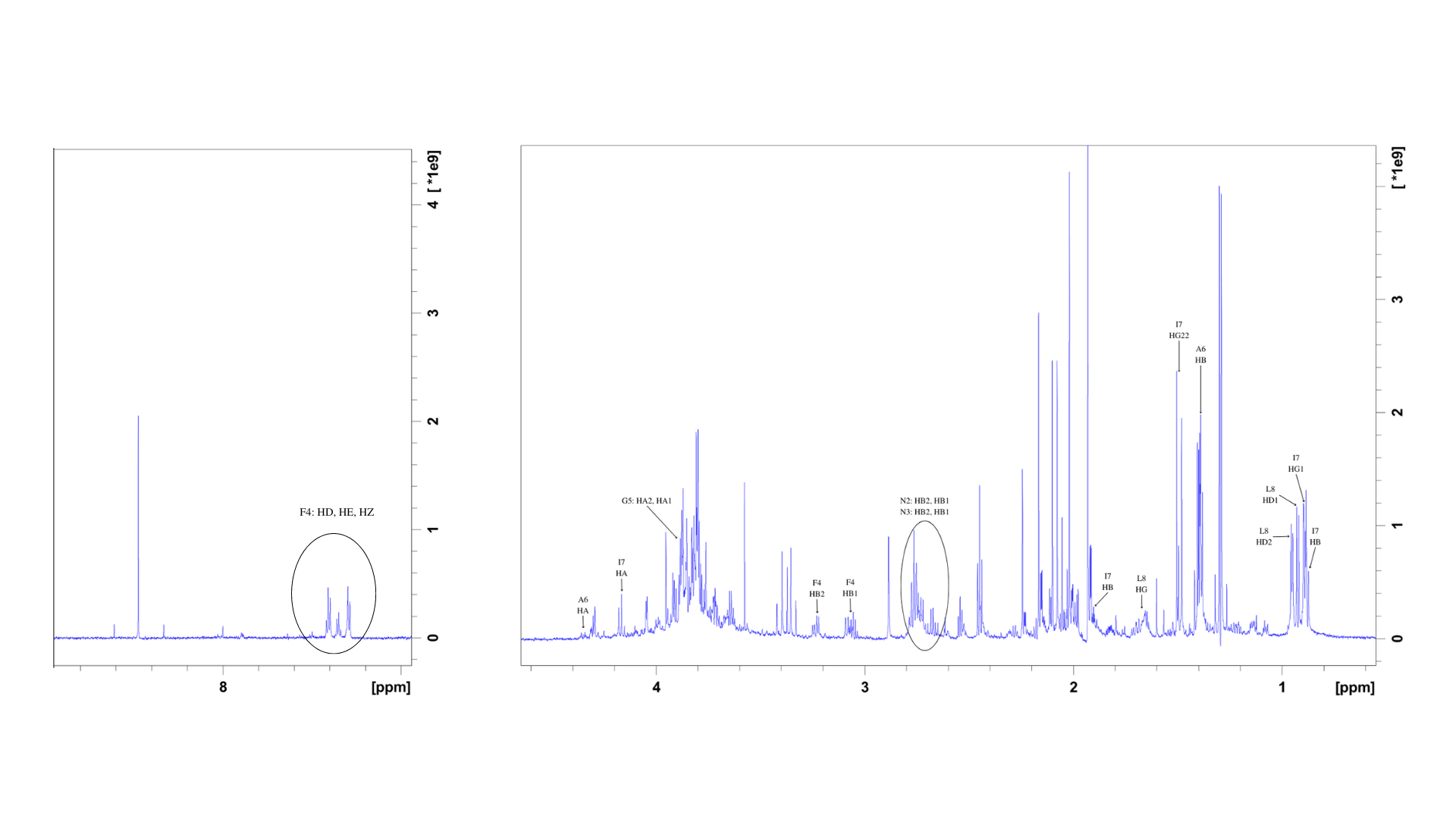
**Figure S10.** Assigned ^1^H NMR spectrum of the peptide hIAPP-F. The shown fragments present the regions of spectrum where signals of the peptide can be find.


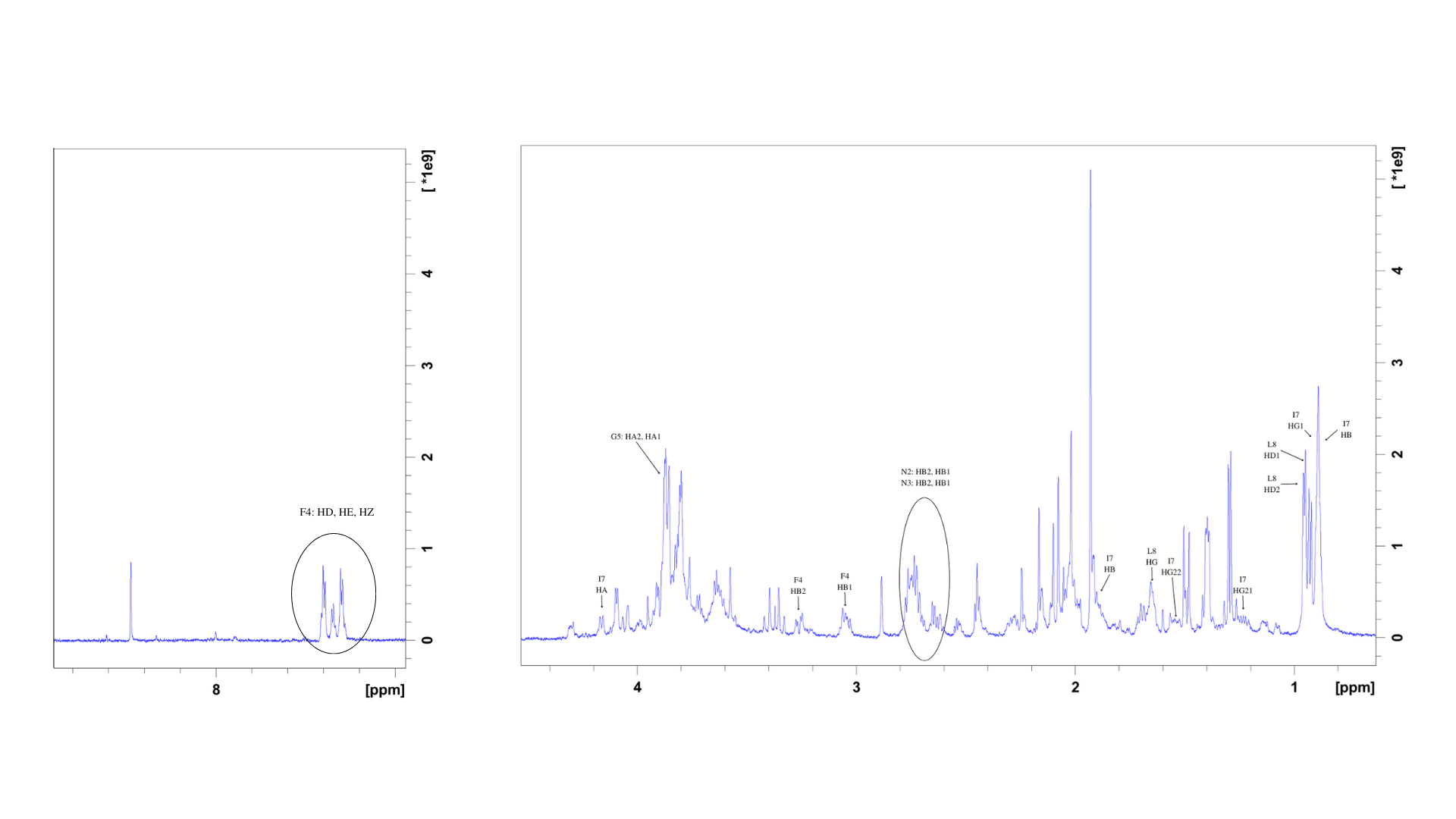
**Figure S11.** Assigned ^1^H NMR spectrum of the peptide pramlintide-M. The shown fragments present the regions of spectrum where signals of the peptide can be find.

**4 Cytotoxicity assay:**

The cytotoxicity assay aimed to evaluate the potential toxicity effects of each peptide on HeLa cells, using the peptides alone and in conjunction with the metal ions Cu(II) and Zn(II). Controls involving Cu(NO_3_)_2_ and ZnCl_2_ were employed (Figure S6) to verify that the concentrations used did not induce toxicity in the cells. Following the analysis of results, the cytotoxicity of hIAPP-F and Pramlintide-M was tested in the presence of 0.5mg/mL Cu(NO_3_)_2_ and ZnCl_2_

**
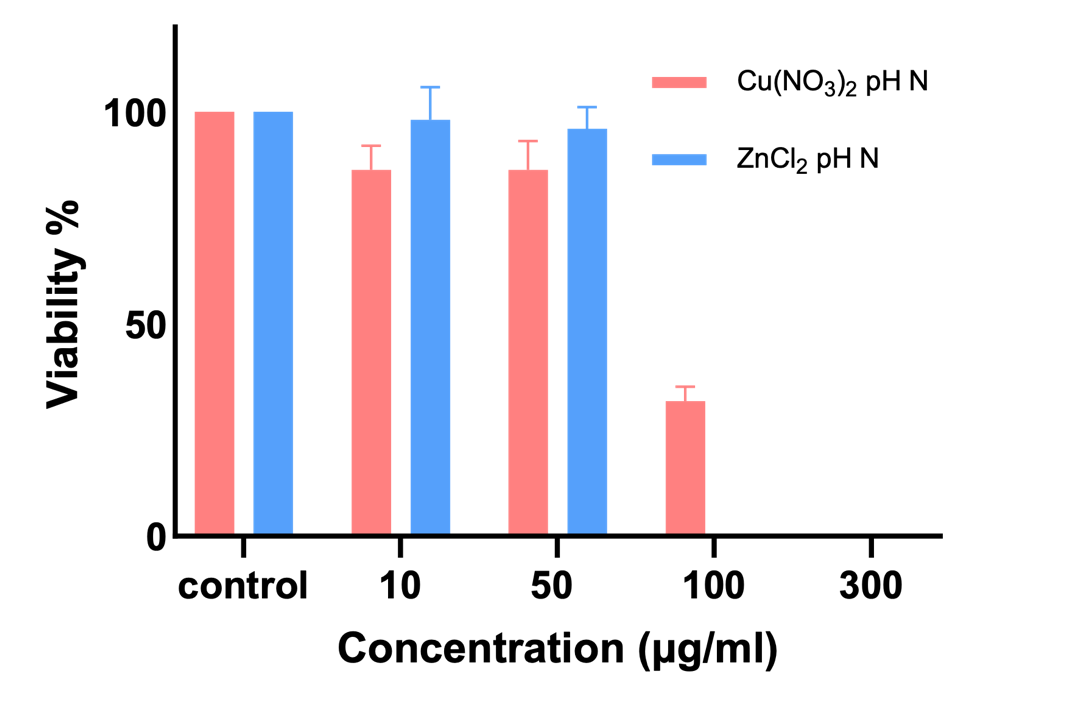
**

**Figure S12**. The cytotoxic effect of different concentrations of Cu(NO_3_)_2_ and ZnCl_2_ on the HeLa cells. Error bars are based on SEM (n = 3)
